# Impacts of COVID-19 on sexual behaviour in Britain: findings from a large, quasi-representative survey (Natsal-COVID)

**DOI:** 10.1136/sextrans-2021-055210

**Published:** 2021-12-16

**Authors:** Catherine H Mercer, Soazig Clifton, Julie Riddell, Clare Tanton, Lily Freeman, Andrew J Copas, Emily Dema, Raquel Bosó Pérez, Jo Gibbs, Wendy Macdowall, Dee Menezes, Mary-Clare Ridge, Chris Bonell, Pam Sonnenberg, Nigel Field, Kirstin R Mitchell

**Affiliations:** 1 Institute for Global Health, University College London, London, UK; 2 NatCen Social Research, London, UK; 3 MRC/CSO Social and Public Health Sciences Unit, University of Glasgow, Glasgow, UK; 4 Faculty of Public Health and Policy, London School of Hygiene and Tropical Medicine, London, UK; 5 Institute for Health Informatics, University College London, London, UK

**Keywords:** COVID-19, sexual behaviour, sexual partners, sexual health

## Abstract

**Objectives:**

Physical restrictions imposed to combat COVID-19 dramatically altered sexual lifestyles but the specific impacts on sexual behaviour are still emerging. We investigated physical and virtual sexual activities, sexual frequency and satisfaction in the 4 months following lockdown in Britain in March 2020 and compared with pre-lockdown.

**Methods:**

Weighted analyses of web panel survey data collected July/August 2020 from a quota-based sample of 6654 people aged 18–59 years in Britain. Multivariable regression took account of participants’ opportunity for partnered sex, gender and age, to examine their independent associations with perceived changes in sexual frequency and satisfaction.

**Results:**

Most participants (86.7%) reported some form of sex following lockdown with physical activities more commonly reported than virtual activities (83.7% vs 52.6%). Altogether, 63.2% reported sex with someone (‘partnered sex’) since lockdown, three-quarters of whom were in steady cohabiting relationships. With decreasing relationship formality, partnered sex was less frequently reported, while masturbation, sex toy use and virtual activities were more frequently reported. Around half of all participants perceived no change in partnered sex frequency compared with the 3 months pre-lockdown, but this was only one-third among those not cohabiting, who were more likely to report increases in non-partnered activities than those cohabiting. Two-thirds of participants perceived no change in sexual satisfaction; declines were more common among those not cohabiting. Relationship informality and younger age were independently associated with perceiving change, often declines, in sexual frequency and satisfaction.

**Conclusions:**

Our quasi-representative study of the British population found a substantial minority reported significant shifts in sexual repertoires, frequency and satisfaction following the introduction of COVID-19 restrictions. However, these negative changes were perceived by some more than others; predominantly those not cohabiting and the young. As these groups are most likely to experience adverse sexual health, it is important to monitor behaviour as restrictions ease to understand the longer term consequences, including for health services.

## Introduction

On 23 March 2020, the UK government announced a strict lockdown to limit SARS-CoV-2 transmission that was effective well into summer 2020 (online supplemental appendix 1). Social distancing rules meant that at no point were people from separate households permitted to have intimate contact unless they were in a ‘support bubble’ in England or an ‘extended household’ in Scotland.^1^ Impacts of these restrictions on individuals’ opportunity for sex with another person (hereon ‘partnered sex’) depended on their circumstances when the lockdown was announced, in particular, whether they had a sexual partner at the time and if so, whether they lived together. These two factors in turn being primarily driven by lifestage.^2 3^ In contrast, opportunities for non-partnered sex (masturbation and virtual sex) may have increased as technology and market forces responded to an anticipated demand^4–7^ as people spent more time at home and online. However, the specific impacts of the COVID-19 pandemic on sexual behaviour are unclear, reflecting how, for example, adherence to physical restrictions was not universal and may have changed over time,^8^ and for those cohabiting, while the opportunity to have sex was theoretically unaffected, inclination to do so may have been altered by lifestyle changes and stress engendered by the pandemic.^9 10^

10.1136/sextrans-2021-055210.supp1 Supplementary data

Understanding the extent to which shifts in sexual behaviour occurred, and who was most affected, are important for public health. Any changes to rates of partner change have implications for STI transmission, even if temporary,^11^ while changes to the frequency of partnered sex are key for interpreting trends in birth rates and abortion.^12^ Despite the pivotal role of sex, there has been relatively little rigorous research examining the direct and indirect effects of COVID-19 on sexual behaviour. We sought to address this evidence gap by investigating: (1) What was the reported prevalence and frequency of physical and virtual sexual activities in Britain following the first national lockdown?; (2) Were there perceived changes in frequency of sexual activity and sexual satisfaction compared with pre-lockdown?; (3) If so, what was the direction and extent of change, and did this vary in the population?

## Methods

Natsal-COVID involved a web panel survey administered 29 July–10 August 2020 by Ipsos MORI. The target sample size was 6500 people residing in Britain, comprising a core sample of 6000 aged 18–59 years and a boost sample of 500 aged 18–29 years. Quotas were used (age, sex, region, social grade) and data weighted to the general population (by age, sex, ethnicity, social grade, sexual identity) to achieve a quasi-representative population sample. Further methodological details—including the sociodemographic and behavioural profile of the achieved sample—have been previously reported.^13^

The questionnaire, available at www.natsal.ac.uk, included questions about participants’ relationship status and if this had changed since lockdown began. Questions about sexual history established whether participants had ever had partnered sex (defined as vaginal, oral, or anal sex with someone of the opposite sex, or any contact involving the genital area with same sex, transgender or non-binary individuals). All participants were asked if they had experienced each of the following physical (1–4) and virtual (5–9) sexual activities since lockdown:

1. Vaginal, anal or oral sex.
2. Other contact with someone’s genital area.
3. Masturbating.
4. Using sex toys (by yourself or with someone else).
5. Messaging via dating apps/online.
6. Sexting (images or recorded videos).
7. Using video or voice calls to interact with someone sexually.
8. Looking at pornography.
9. Paying for online sexual services (eg, live streaming).

Participants were asked how frequently they had engaged in each activity since the start of lockdown and whether this was more or less compared with the 3 months before lockdown. Participants who reported ever having partnered sex (hereon ‘the sexually experienced’) were asked if they perceived changes since lockdown in their frequency of sex and satisfaction with their sex life.

We did complex survey analysis using Stata (V.15) to obtain population estimates. We first present the proportion and frequency of (1) reporting each of the sexual activities (above) since lockdown, and (2) perceiving change in the frequency of each activity compared with the 3 months pre-lockdown. Estimates are presented for five groups that reflect participants’ opportunity for partnered sex and relationship formality given the assumed importance of these factors on individuals’ sexual behaviour during the lockdown period:

1. No partnered sex ever.
2. No partnered sex since lockdown.
3. Partnered sex since lockdown but no steady relationship.
4. Partnered sex since lockdown in a steady non-cohabiting relationship.
5. Partnered sex since lockdown in a steady cohabiting relationship (online supplemental appendix 2).

The denominator for these analyses was the total sample reflecting our aim to consider sexual activity for the population as a whole. However, we limited the denominator to the sexually experienced when we examined participants’ perceptions of change in (1) frequency of sex and (2) satisfaction with their sex life. For each outcome, we used multivariable logistic regression to calculate ORs for reporting an increase, and a decrease (each relative to no change) for partnered sex/relationship status group, age group, and gender, and then calculated adjusted ORs to examine their independent associations. Given that changes in sexual frequency may lead to changes in sexual satisfaction,^14 15^ we additionally adjusted for the former to assess the impact on any associations observed with sexual satisfaction.

We also present our analyses stratified by gender (online supplemental appendices 4a, b and 6a, b) and age group (online supplemental appendices 5a–d and 7a–d) reflecting the importance of these demographics for the context of sexual activity.

## Results

Almost two-thirds of the achieved sample (4073 of 6654) reported partnered sex in the 4 months following lockdown (online supplemental appendix 2). Most of these (3453 of 4073) were in a steady relationship, and of these, the majority lived with their partner (3094 of 3453). One-third of all participants (2288 of 6654) reported no partnered sex since lockdown, with one-third of these (743 of 2288) reporting *never* having partnered sex. Most reporting no partnered sex since lockdown described themselves as not in a steady relationship (68.7%, table 1), while 25.7% were in a steady cohabiting relationship. Of all those not in a steady relationship, only around one-quarter reported partnered sex since lockdown. This compares with almost three-quarters of those in a steady *non*-cohabiting relationship and 81.0% of those in a steady cohabiting relationship.

**Table 1:** Cross-tabulation of experience of partnered sex by relationship status since lockdown

	Denominators (unweighted, weighted)*	Relationship status since lockdown							
		Not in a steady relationship		Steady non-cohabiting relationship		Steady cohabiting relationship		All	
		Row %	Column %	Row %	Column %	Row %	Column %	Row %	Column %
Experience of partnered sex									
No partnered sex ever	743, 762	78.6	25.4	3.3	5.5	18.2	3.6	**100.0**	**11.5**
Partnered sex ever but not since lockdown	1545, 1579	68.7	46.0	5.6	19.5	25.7	10.6	**100.0**	**23.7**
Partnered sex since lockdown	4073, 4012	14.5	24.4	8.1	71.7	77.5	81.0	**100.0**	**60.3**
Not known	293, 300	32.9	4.2	4.9	3.2	62.2	4.9	**100.0**	**4.5**
Total	**6654, 6654**	**35.5**	**100.0**	**6.8**	**100.0**	**57.7**	**100.0**	**100.0**	**100.0**
Denominators (unweighted, weighted)*		2383, 2359		494, 453		3777, 3842		6654, 6654	

Table created by the authors.

*Denominators: all respondents.

The reporting of partnered sex and relationship status was broadly similar by gender (online supplemental appendices 3a, b). However, although men were as likely as women to report partnered sex since lockdown, they were less likely to be in a steady relationship. There was greater variation by age (online supplemental appendices 3a, b). The proportion reporting never having partnered sex was highest among the young: around one-quarter of those aged under 25 years reducing to around 1 in 10 of those aged 25 years and older. Conversely, the proportion reporting partnered sex but not since lockdown was higher in older people (one in three of those 45–59 years vs one in six aged under 35 years). Among all participants, the proportion who reported partnered sex since lockdown with a steady cohabiting partner increased with age from one-quarter of those under 25 years to over half of those 25–44 years, but fell to just under half of those 45 years and older.

Of all participants, 86.7% reported some type of sexual activity during the first 4 months of lockdown (table 2), including over half of those reporting no partnered sex (either ever or since lockdown). Physical activities were more commonly reported than virtual activities (83.7% vs 52.6%). However, with decreasing relationship formality, partnered sex was less often reported while masturbation, sex toy use and virtual activities were more so.

**Table 2:** Type and frequency of sexual activity reported since lockdown stratified by experience of partnered sex and relationship status

Reported partnered sex since lockdown	No		Yes			All % (CI)	P value
Sexual activity since lockdown	Among those reporting never having partnered sex % (CI)	Among those reporting partnered sex ever but not since lockdown % (CI)	Among those not in a steady relationship % (CI)	Among those in a steady, non-cohabiting relationship % (CI)	Among those in a steady, cohabiting relationship % (CI)		
**% reporting any sexual activity**	53.8 (49.5 to 57.9)	65.4 (62.8 to 68.0)	100.0	100.0	100.0	86.7 (85.7 to 87.6)	<0.0001
Denominators*	*639, 648*	*1487, 1518*	*620, 576*	*359, 325*	*3094, 3111*	*6199, 6178*	
**Physical sexual activity since lockdown**							
**% reporting any physical sexual activity†**	42.0 (38.0 to 46.2)	58.3 (55.6 to 61.0)	100.0	100.0	100.0	83.7 (82.7 to 84.7)	<0.0001
Denominators*	*642, 655*	*1458, 1489*	*620, 576*	*359, 325*	*3094, 3111*	*6173, 6156*	
**% reporting any partnered sex‡**	0	0	100.0	100.0	100.0	63.2 (61.9 to 64.4)	<0001
Denominators*	*762, 743*	*1579, 1545*	*576, 620*	*325, 359*	*3111, 3094*	*6354, 6361*	
**% reporting any vaginal, oral, and/or anal sex**	0	0	91.7 (88.8 to 93.9)	97.4 (94.5 to 98.8)	97.7 (97.0 to 98.2)	61.7 (60.36 to 63.0)	<0.0001
Denominators*	*687, 703*	*1545, 1579*	*618, 573*	*358, 325*	*3088, 3107*	*6296, 6287*	
Of those who did, frequency since lockdown							
Less than weekly	–	–	53.2 (48.6 to 57.7)	47.1 (41.5 to 52.8)	40.7 (38.9 to 42.6)	42.9 (41.3 to 44.6)	<0.0001
At least once a week	–	–	46.8 (42.3 to 51.4)	52.9 (47.3 to 58.5)	59.3 (57.4 to 61.1)	57.1 (55.4 to 58.7)	
*Denominators§*	*–*	*–*	*572, 526*	*349, 316*	*3017, 3035*	*3938, 3877*	
**% reporting other contact with someone’s genital area**	0	0	86.8 (83.4 to 89.5)	86.0 (81.4 to 89.5)	85.6 (84.2 to 86.9)	54.3 (52.9 to 55.6)	0.80
Denominators*	*690, 708*	*1545 1579*	*603, 558*	*357, 324*	*3041, 3055*	*6236, 6224*	
Of those who did, frequency since lockdown							
Less than weekly	–	–	55.4 (50.6 to 60.1)	43.1 (37.3 to 49.1)	36.5 (34.6 to 38.5)	39.8 (38.0 to 41.5)	<0.0001
At least once a week	–	–	44.6 (39.9 to 49.4)	56.9 (50.9 to 62.7)	63.5 (61.5 to 65.4)	60.2 (58.5 to 62.0)	
*Denominators§*	*–*	*–*	*528, 484*	*312, 278*	*2630, 2616*	*3470, 3378*	
**% reporting masturbation**	39 (35.1 to 43.1)	56.9 (54.2 to 59.6)	77.5 (73.4 to 81.1)	69.0 (63.5 to 74.0)	57.1 (55.2 to 58.9)	57.4 (56.1 to 58.8)	<0.0001
Denominators*	*673, 692*	*1460, 1491*	*595, 548*	*343, 310*	*3009, 3027*	*6080, 6068*	
Of those who did, frequency since lockdown							
Less than weekly	29.4 (23.7 to 35.8)	29.1 (25.9 to 32.4)	28.0 (23.7 to 32.7)	39.9 (33.2 to 46.9)	36.7 (34.3 to 39.1)	33.4 (31.8 to 35.1)	<0.0001
At least once a week	70.6 (64.2 to 76.3)	70.9 (67.6 to 74.1)	72.0 (67.3 to 76.3)	60.2 (53.1 to 66.8)	63.3 (60.9 to 65.7)	66.6 (64.9 to 68.2)	
*Denominators§*	*277, 270*	*883, 849*	*479, 425*	*239, 214*	*1790, 1727*	*3668, 3484*	
**% reporting using sex toys (by yourself or with someone else**)	4.6 (3.3 to 6.4)	11.9 (10.3 to 13.7)	46.9 (42.6 to 51.4)	37.8 (32.5 to 43.5)	28.5 (26.8 to 30.2)	23.7 (22.6 to 24.8)	<0.0001
Denominators*	*704, 724*	*1518, 1553*	*601, 554*	*349, 315*	*3050, 3072*	*6222, 6218*	
Of those who did, frequency since lockdown							
Less than weekly	46.0 (29.9 to 62.9)	53.9 (46.6 to 61.2)	41.4 (35.3 to 47.8)	60.4 (51.1 to 69.0)	53.6 (50.2 to 57.1)	51.9 (49.2 to 54.6)	0.0030
At least once a week	54.0 (37.1 to 70.1)	46.1 (38.9 to 53.5)	58.6 (52.2 to 64.7)	39.6 (31.0 to 49.0)	46.4 (42.9 to 49.9)	48.1 (45.5 to 50.8)	
*Denominators§*	*40, 33‡‡*	*215, 185*	*300, 260*	*139, 119*	*943, 876*	*1637, 1473*	
**Virtual sexual activities since lockdown**							
**% reporting any virtual sexual activity¶**	40.9 (37.0 to 45.0)	51.0 (48.3 to 53.7)	84.3 (80.8 to 87.2)	68.4 (63.0 to 73.4)	48.4 (46.5 to 50.3)	52.6 (51.2 to 53.9)	<0.0001
Denominators*	*674, 690*	*1507, 1539*	*610, 565*	*356, 322*	*3045, 3063*	*6192, 6180*	
% reporting any virtual sexual activity excluding looking at pornography**	19.4 (16.4 to 22.7)	25.6 (23.4 to 28.0)	73.7 (69.6 to 77.4)	54.5 (48.8 to 60.0)	21.0 (19.5 to 22.6)	28.5 (27.3 to 29.7)	<0.0001
Denominators*	*687, 705*	*1527, 1561*	*613, 569*	*356, 322*	*3068, 3086*	*6251, 6243*	
**% reporting messaging via dating apps/online**	16.1 (13.4 to 19.2)	21.6 (19.5 to 23.9)	59.7 (55.3 to 64.0)	38.4 (33.0 to 44.0)	13.7 (12.5 to 15.1)	21.4 (20.3 to 22.5)	<0.0001
Denominators*	*702, 719*	*1537, 1571*	*608, 564*	*357, 323*	*3066, 3085*	*6270, 6262*	
Of those who did, frequency since lockdown							
Less than weekly	53.8 (44.1 to 63.2)	41.1 (35.7 to 46.8)	28.4 (23.5 to 33.9)	19.6 (12.9 to 28.7)	27.9 (23.5 to 32.8)	32.9 (30.2 to 35.6)	<0.0001
At least once a week	46.2 (36.8 to 55.9)	58.9 (53.2 to 64.3)	71.6 (66.2 to 76.5)	80.4 (71.3 to 87.1)	72.1 (67.2 to 76.5)	67.2 (64.4 to 69.8)	
*Denominators§*	*127, 115*	*367, 340*	*374, 337*	*140, 124*	*442, 424*	*1450, 1339*	
**% reporting sexting (images or recorded videos**)	4.8 (3.4 to 6.8)	9.6 (8.2 to 11.2)	49.6 (45.2 to 53.9)	36.0 (30.8 to 41.5)	15.2 (13.9 to 16.6)	16.8 (15.8 to 17.8)	<0.0001
Denominators*	*707, 726*	*1532, 1567*	*611, 568*	*357, 324*	*3080, 3099*	*6287, 6284*	
Of those who did, frequency since lockdown							
Less than weekly	64.7 (47.5 to 78.8)	51.2 (42.9 to 59.5)	44.8 (38.7 to 51.1)	55.3 (45.9 to 64.3)	45.8 (41.1 to 50.6)	48.0 (44.8 to 51.2)	0.080
At least once a week	35.3 (21.2 to 52.5)	48.8 (40.5 to 57.1)	55.2 (48.9 to 61.3)	44.7 (35.7 to 54.1)	54.2 (49.4 to 58.9)	52.0 (48.8 to 55.2)	
*Denominators§*	*41, 35‡‡*	*169, 150*	*314, 281*	*131, 117*	*502, 472*	*1157, 1055*	
**% reporting using video or voice calls to interact with someone sexually**	3.2 (2.0 to 5.1)	5.8 (4.7 to 7.2)	43.9 (39.6 to 48.3)	31.2 (26.2 to 36.7)	11.5 (10.3 to 12.7)	13.0 (12.2 to 14.0)	<0.0001
Denominators*	*709, 728*	*1532, 1567*	*611, 565*	*356, 323*	*3076, 3094*	*6284, 6276*	
Of those who did, frequency since lockdown							
Less than weekly	–	54.6 (43.7 to 65.0)	45.3 (38.6 to 52.2)	47.8 (37.9 to 58.0)	44.1 (38.7 to 49.7)	46.8 (43.1 to 50.5)	0.19
At least once a week	–	45.5 (35.0 to 56.4)	54.7 (47.8 to 61.4)	52.2 (42.0 to 62.2)	55.9 (50.3 to 61.3)	53.3 (49.5 to 56.9)	
*Denominators§*	*21, 23††*	*92, 92*	*254, 248*	*108, 101*	*361, 355*	*836, 818*	
**% reporting looking at pornography**	29.6 (26.0 to 33.4)	41.1 (38.5 to 43.7)	67.6 (63.3 to 71.6)	49.5 (43.9 to 55.1)	42.4 (40.5 to 44.3)	43.2 (41.9 to 44.6)	<0.0001
Denominators*	*689, 707*	*1497, 1529*	*599, 553*	*352, 320*	*3048, 3067*	*6185, 6176*	
Of those who did, frequency since lockdown							
Less than weekly	33.6 (26.8 to 41.1)	29.0 (25.4 to 32.9)	38.5 (33.4 to 44.0)	38.2 (30.6 to 46.4)	41.5 (38.7 to 44.4)	37.3 (35.4 to 39.3)	<0.0001
At least once a week	66.4 (58.9 to 73.3)	71.0 (67.1 to 74.6)	61.5 (56.0 to 66.6)	61.8 (53.7 to 69.4)	58.5 (55.6 to 61.3)	62.7 (60.7 to 64.6)	
*Denominators§*	*207, 209*	*659, 628*	*411, 374*	*178, 159*	*1337, 1300*	*2792, 2669*	
**% reporting paying for online sexual services (eg, live streaming**)	0.9 (0.4 to 1.9)	0.7 (0.4 to 1.3)	23.8 (20.2 to 27.9)	10.2 (7.2 to 14.3)	6.9 (6.0 to 8.0)	6.4 (5.7 to 7.0)	<0.0001
Denominators*	*716, 736*	*1542, 1576*	*608, 563*	*357, 323*	*3079, 3097*	*6302, 6294*	
Of those who did, frequency since lockdown							
Less than weekly	–	–	45.5 (36.3 to 55.0)	34.3 (19.8 to 52.3)	40.2 (33.4 to 47.5)	42.7 (37.5 to 48.0)	0.18
At least once a week	–	–	54.5 (45.0 to 63.7)	65.8 (47.7 to 80.2)	59.8 (52.5 to 66.7)	57.4 (52.0 to 62.6)	
*Denominators§*	*7, 6††*	*12, 11††*	*131, 134*	*35, 33‡‡*	*216, 215*	*401, 400*	

Table created by the authors.

*Denominators (unweighted, weighted): all respondents.

†Reported at least one of the following activities since lockdown: vaginal, anal or oral sex, other contact with someone’s genital area, masturbating, using sex toys (by yourself or with someone else).

‡Reported at least one of the following partnered activities since lockdown: vaginal, anal or oral sex, other contact with someone’s genital area.

§Denominators (unweighted, weighted): all respondents (who reported the activity in lockdown).

¶Reported at least one of the following since lockdown: messaging via dating apps/online, sexting (images or recorded videos), using video or voice calls to interact with someone sexually, looking at pornography, paying for online sexual services (eg, live streaming).

**Reported at least one of the following since lockdown: Messaging via dating apps/online, sexting (images or recorded videos), using video or voice calls to interact with someone sexually, paying for online sexual services (eg, live streaming).

††Unweighted denominator <30 so estimates not shown due to small denominator.

‡‡Unweighted denominator. Results should be interpreted with caution due to small denominator.

The proportion reporting virtual activities since lockdown was largely driven by pornography use during this time, reported by 43.2% overall, but men were much more likely than women to do so (65.1% vs 21.1%, online supplemental appendices 4a, b). As with masturbation and sex toy use, pornography use declined with increasing relationship formality, but there was little difference in frequency by relationship status. Other virtual activities were less commonly reported at around one in five or fewer.

**Figure 1:**
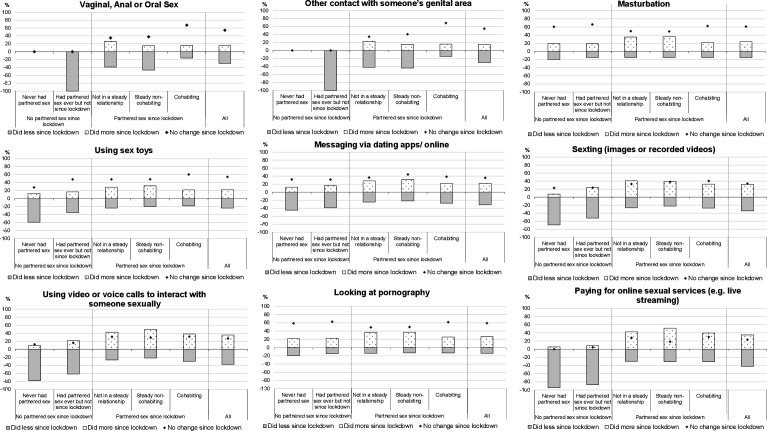
Extent and direction of perceived change in frequency of particular physical and virtual sexual activities compared to pre-lockdown, stratified by experience of partnered sex and relationship status since lockdown.

Figure 1 shows the extent and direction of perceived change in frequency of each physical and virtual sexual activity. Overall, just over half of participants perceived no change in their frequency of partnered physical activities but considerable differences exist by relationship status. Cohabiting participants were twice as likely as those not cohabiting to report no change. Among those who did report change, cohabitees were as likely to perceive increases as decreases in frequency, but those not cohabiting were far more likely to report decreases, especially those in steady relationships.

Of all participants, 61.1% reported no change in frequency of masturbation compared with pre-lockdown. However, among those who did, increases were more common than decreases (23.7% vs 15.3%), a pattern evident specifically for those reporting partnered sex and those not cohabiting. These patterns were also observed for sex toy use.

Whereas most participants reported no change in frequency of physical activities, the opposite was true for virtual activities: around two-thirds reported change with increases as likely as decreases (figure 1). Although the numbers reporting virtual activities were relatively small, it seems that changes—specifically declines—were more common among those reporting no partnered sex (either ever or since lockdown). Pornography use however was different: 59.3% reported no change and where change was reported then increases were almost twice as likely as decreases (26.5% vs 14.2%). Increases were most likely among participants reporting partnered sex since lockdown, and again, especially those not cohabiting.

The extent and direction of perceived change in sexual frequency overall (online supplemental appendix 8) were similar to what was observed for partnered physical sexual activities (figure 1)—at least for those reporting partnered sex since lockdown. Change was most likely to be perceived by those not cohabiting, for whom declines in frequency were far more common than increases. Of those reporting no partnered sex since lockdown, two-thirds perceived no change in (overall) sexual frequency, but the remaining one-third all described this as a decline.

Perceptions of changes in sexual frequency were similar by gender but striking differences were observed by age. Younger people were far more likely to perceive change (60.7% of those 18–24 years reducing to 32.1% of those 45–59 years), and specifically declines (40.1% vs 25.4%, respectively). However, younger people were also more likely to perceive increases in frequency relative to older people (20.6% vs 6.7%, respectively).

Perceived changes in sexual satisfaction were less commonly reported overall than perceived changes in sexual frequency (36.9% vs 44.5%; online supplemental appendix 9). Participants were more likely to report ‘mostly negative’ than ‘mostly positive’ changes in their satisfaction (23.3% vs 14.0%). The proportion of those reporting partnered sex since lockdown who perceived ‘mostly negative’ changes increased with relationship informality from 16.5% of those cohabiting to 31.0% of those with no steady partner, which was as high as observed for those reporting no partnered sex since lockdown (32.7%). Only 2.5% of those reporting no partnered sex since lockdown perceived ‘mostly positive’ changes in satisfaction in contrast to around one in five of all those who reported partnered sex since lockdown, regardless of relationship status.

Men were slightly more likely to perceive change in sexual satisfaction than women and for this to be ‘mostly negative’. However, as with sexual frequency, larger differences in perceived satisfaction were observed by age, being largest among younger participants (57.4% of those 18–24 years vs 26.3% of those 45–59 years) and more likely to be ‘mostly negative’(31.5% vs 18.8%, respectively).

**Figure 2:**
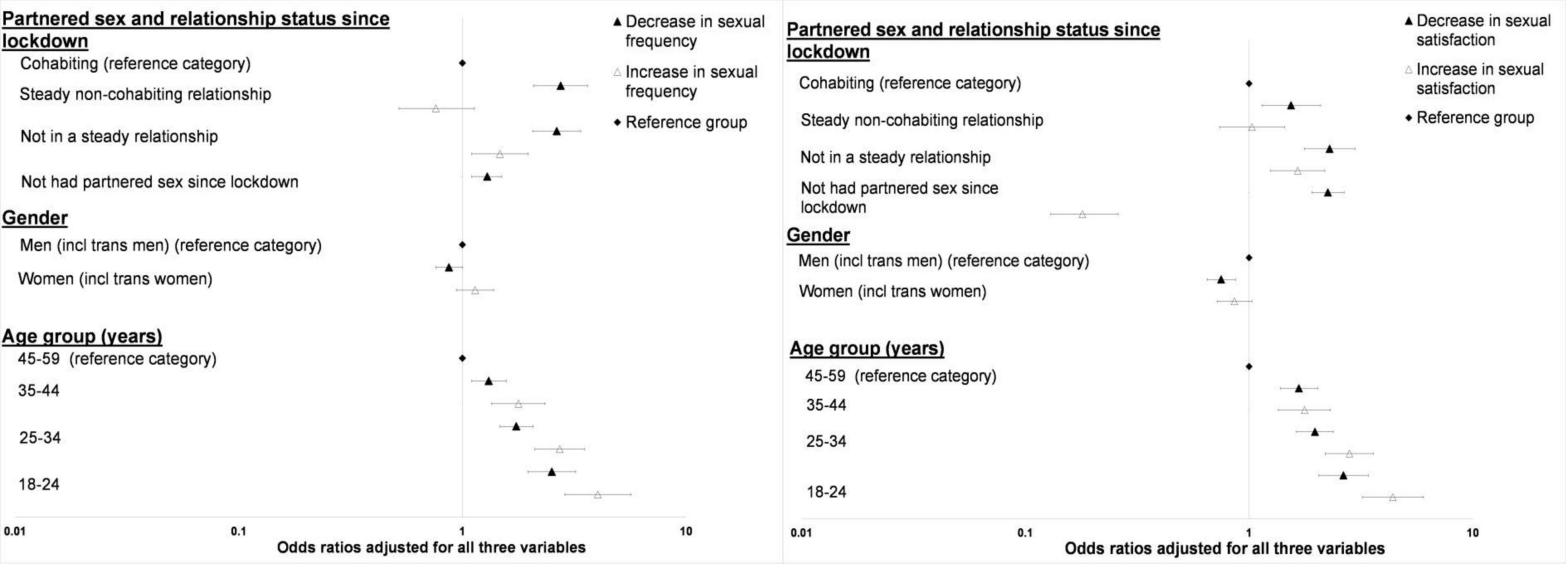
Forest plots showing the adjusted odds ratios for perceiving a decrease and an increase (each relative to no change) in (A) sexual frequency compared to pre-lockdown, and (B) sexual satisfaction compared to pre-lockdown, according to partnered sex/relationship status, gender, and age-group.

In multivariable analyses (figure 2A, B), gender was independently associated with perceived changes in sexual satisfaction (but not frequency); men faring worse than women. For both outcomes, age was independently associated with perceiving change—and in either direction. Similarly, participants’ experience of partnered sex and relationship status since lockdown remained independently associated with both outcomes. Taking account of perceived change in frequency had little impact on these independent associations, although the magnitude of the adjusted ORs for perceiving ‘mostly negative’ change reduced for those not cohabiting (online supplemental appendix 9).

## Discussion

Natsal-COVID suggests most people in Britain were sexually active following the initial national lockdown in March 2020. While reporting of physical partnered sex increased with relationship formality, non-partnered and virtual activities were more commonly reported by those not in steady relationships. Sexual activity at a population level appears largely unaffected by the COVID-19 restrictions, reflecting how most of the British population are in cohabiting relationships.^2 3^ However, a substantial minority perceived sizeable changes following the initial lockdown. Declines in frequency were perceived most commonly by those not living with a partner but who reported partnered sex since lockdown. Declines in satisfaction were perceived most commonly by those with no steady partner but who reported sex since lockdown and those reporting no partnered sex since lockdown. Age was strongly and independently associated with perceived changes in sexual frequency and satisfaction, with younger people more likely to report both increases and decreases, suggesting influences independent of lockdown.

To our knowledge, this is the largest and most comprehensive study of sexual behaviour since the beginning of the COVID-19 pandemic in Britain and one of the most comprehensive worldwide. Strengths include quasi-representative sampling using an established web panel, which has been shown to be more representative of the general population than convenience sampling.^16^ However, this methodology has limitations. Estimates from online panels should be treated with caution given likely selection and response biases.^17^ By using online methods, we excluded those without internet access, which in the UK in 2019 was 13% of those aged 16 years and over, and higher among those in lower social grades.^18^ However, during the COVID-19 pandemic, ‘gold standard’ data collection, for example, probability sampling with in-person interviewing, was not possible due to physical distancing and rapid data collection requirements. Elsewhere,^13^ we compared our sample with external data sources to assess measurable biases and found some differences in marital status, education level and health status. There is no contemporary external comparator for the sexual behaviour data, and previous research has shown that web panel estimates for sexual behaviour differ to those achieved through probability sampling.^17^ Comparisons with Britain’s last Natsal survey show that while the Natsal-COVID sample was less likely to report partnered sex ever, similarities exist in reported partner numbers and same-sex experience among those sexually active.^13^ While virtual sexual activities were less commonly reported than physical activities, it is plausible that using an online sample resulted in higher reporting of virtual activities—a hypothesis to be addressed by the next decennial survey, Natsal-4, which includes questions on both virtual and physical sexual activities.

The lack of pre-pandemic baseline data limited our ability to quantify change. Our findings on perceived change are subject to recall bias and social desirability bias, which may have been greater for activities prohibited in lockdown.^19 20^ Our survey was fielded when restrictions were easing (online supplemental appendix 1), which may have affected participants’ reporting, for example, possible greater willingness to report previously prohibited activities, or a more favourable take on changes to sex lives during lockdown.

Natsal-COVID sought to define sex broadly, and also change in overall sexual activity, but the lay perspective often considers sex as primarily partnered, and typically penetrative intercourse.^21^ This seems to be how many in Natsal-COVID responded to the question on perceived change in frequency as the patterns observed mirrored those specifically for physical partnered activities among those reporting partnered sex. Therefore, while we sought to capture change in sexual activity overall, this may not have been how some participants answered it.

Our results clearly show that changes in frequency were independently associated with both relationship status and age. Studies accounting for relationship status are rare but have generally shown, as we did, that not living with a partner was a key determinant of change; typically but not always a deterioration.^22^ Others have reported that declines in frequency were common in young people,^23^ which we show is not fully explained by relationship status: it seems lifestage itself is important.

Our findings that only one-quarter of participants not in steady relationships reported partnered sex since lockdown, and among those that did, this was perceived as having declined, have important consequences for public health. Those not in steady relationships are most likely to have new and casual partners,^24 25^ both key STI risk behaviours, so it follows that STI transmission may have reduced following the initial lockdown.^26^ However, as restrictions ease, it is likely that there will be an uptick in partner change rates and therefore STI transmission and unintended pregnancy, with implications for the demand for sexual and reproductive health (SRH) services. Lockdown restrictions may have meant a delay to sexual debut for some young people. Coupled with lockdown limiting young people’s access to relationship and sex education delivered through schools, community organisations and peer discussion, it is plausible that this cohort may be more vulnerable to adverse circumstances when they do experience sexual debut, with implications for their subsequent sexual well-being.^27^

We found that the majority of those with a steady but non-cohabiting partner reported partnered sex since lockdown despite this implying non-adherence to physical restrictions. This suggests that virtual partnered and non-partnered sexual activities, as activities unaffected by lockdown restrictions, were insufficient substitutes for intimacy among individuals living apart from their partner. While more commonly reported by those in non-cohabiting relationships, cohabiting participants did so too suggesting that virtual and non-partnered sexual activities may play both complementary and compensatory roles for some. The broadening of sexual repertoires, including through the adoption, or at least trial, of virtual activities experienced by some because of lockdown, further fuels the rapid technological and social changes underway prior to the pandemic, with technologically mediated ways of being sexually intimate expanding in scope and social acceptability. Indeed, studies are already reporting increases, for example, in live-cam streaming^5^ and pornography use,^6 7^ and retail data are claiming increases since lockdown in sex toy sales.^28^ The potential shift in sexual repertoires, specifically the balance between in-person and virtual sexual activities, also has implications for public health; the benefit of virtual activity in terms of reduced STI and pregnancy risk may be offset by detrimental impacts on sexual satisfaction and well-being. Sexual activity and the context in which this occurs therefore require monitoring through the pandemic and beyond to ascertain whether the observed changes simply represent a temporary adjustment to circumstances or are a longer term trend, especially for those disproportionately affected by poor SRH.

**Key messages**

- Many studies investigating the effects of COVID-19 on sexual behaviour have used small, clinic-based and/or convenience samples which are not representative of the general population.
- Natsal-COVID involved a large (N=6654) web panel survey with quota sampling and statistical weighting of the data, making the findings quasi-representative of the British general population.
- Most people reported some form of sex in the 4 months following lockdown, with only a minority perceiving changes, usually declines, in sexual frequency and/or satisfaction.
- Inequalities were observed in perceiving changes, with implications for the demand for, and provision of, sexual and reproductive health services, and tracking trends therein.

## Data Availability

The data are available from the UK Data Service (study number: 8865) alongside other datasets from Natsal.
