# Supplementary material for: Impacts of COVID-19 on sexual behaviour in Britain: findings from a large, quasi-representative survey (Natsal-COVID)
Source: Sex Transm Infect. 2021 Dec 14:sextrans-2021-055210. doi: 10.1136/sextrans-2021-055210 (PMC8687784; doi:10.1136/sextrans-2021-055210)
Supplement: Supplementary file 1 [file sextrans-2021-055210supp001.pdf]

Online supplementary appendices:

Appendix 1: Timeline of Natsal-COVID study and COVID-19 restrictions in Britain.

Appendix 2: Flowchart of the sample distribution according to experience of partnered sex and relationship status since lockdown.

Appendix 3a: Distribution of experience of partnered sex and relationship status since lockdown stratified by age-group: Men.

Appendix 3b: Distribution of experience of partnered sex and relationship status since lockdown stratified by age-group: Women.

Appendix 4a: Type and frequency of sexual activity reported since lockdown stratified by experience of partnered sex and relationship status since lockdown: Men.

Appendix 4b: Type and frequency of sexual activity reported since lockdown stratified by experience of partnered sex and relationship status since lockdown: Women.

Appendix 5a: Type and frequency of sexual activity reported since lockdown stratified by experience of partnered sex and relationship status since lockdown: Age-group 18-24 years.

Appendix 5b: Type and frequency of sexual activity reported since lockdown stratified by experience of partnered sex and relationship status since lockdown: Age-group 25-34 years.

Appendix 5c: Type and frequency of sexual activity reported since lockdown stratified by experience of partnered sex and relationship status since lockdown: Age-group 35-44 years.

Appendix 5d: Type and frequency of sexual activity reported since lockdown stratified by experience of partnered sex and relationship status since lockdown: Age-group 45-59 years.

Appendix 6a: Extent and direction of perceived change in frequency of particular physical and virtual sexual activities compared to pre-lockdown, stratified by experience of partnered sex and relationship status since lockdown: Men.

Appendix 6b: Extent and direction of perceived change in frequency of particular physical and virtual sexual activities compared to pre-lockdown, stratified by experience of partnered sex and relationship status since lockdown: Women

Mercer CH, *et al. Sex Transm Infect* 2021;0:1–9. doi: 10.1136/sextrans-2021-055210

Appendix 7a: Extent and direction of perceived change in frequency of particular physical and virtual sexual activities compared to pre-lockdown, stratified by experience of partnered sex and relationship status since lockdown: Age-group 18-24 years.

Appendix 7b: Extent and direction of perceived change in frequency of particular physical and virtual sexual activities compared to pre-lockdown, stratified by experience of partnered sex and relationship status since lockdown: Age-group 25-34 years.

Appendix 7c: Extent and direction of perceived change in frequency of particular physical and virtual sexual activities compared to pre-lockdown, stratified by experience of partnered sex and relationship status since lockdown: Age-group 35-44 years.

Appendix 7d: Extent and direction of perceived change in frequency of particular physical and virtual sexual activities compared to pre-lockdown, stratified by experience of partnered sex and relationship status since lockdown: Age-group 45-59 years.

Appendix 8: Percentages, crude and adjusted odds ratios (OR) for perceiving a decrease and an increase (each relative to no change) in sexual frequency compared to pre-lockdown, according to experience of partnered sex/relationship status since lockdown, gender, and age-group

Appendix 9: Percentages, crude and adjusted odds ratios for perceiving a decrease and an increase (each relative to no change) in sexual satisfaction compared to pre-lockdown, according to experience of partnered sex/relationship status, gender, and age-group

Appendix 1: Timeline of Natsal-COVID study and COVID-19 restrictions in Britain

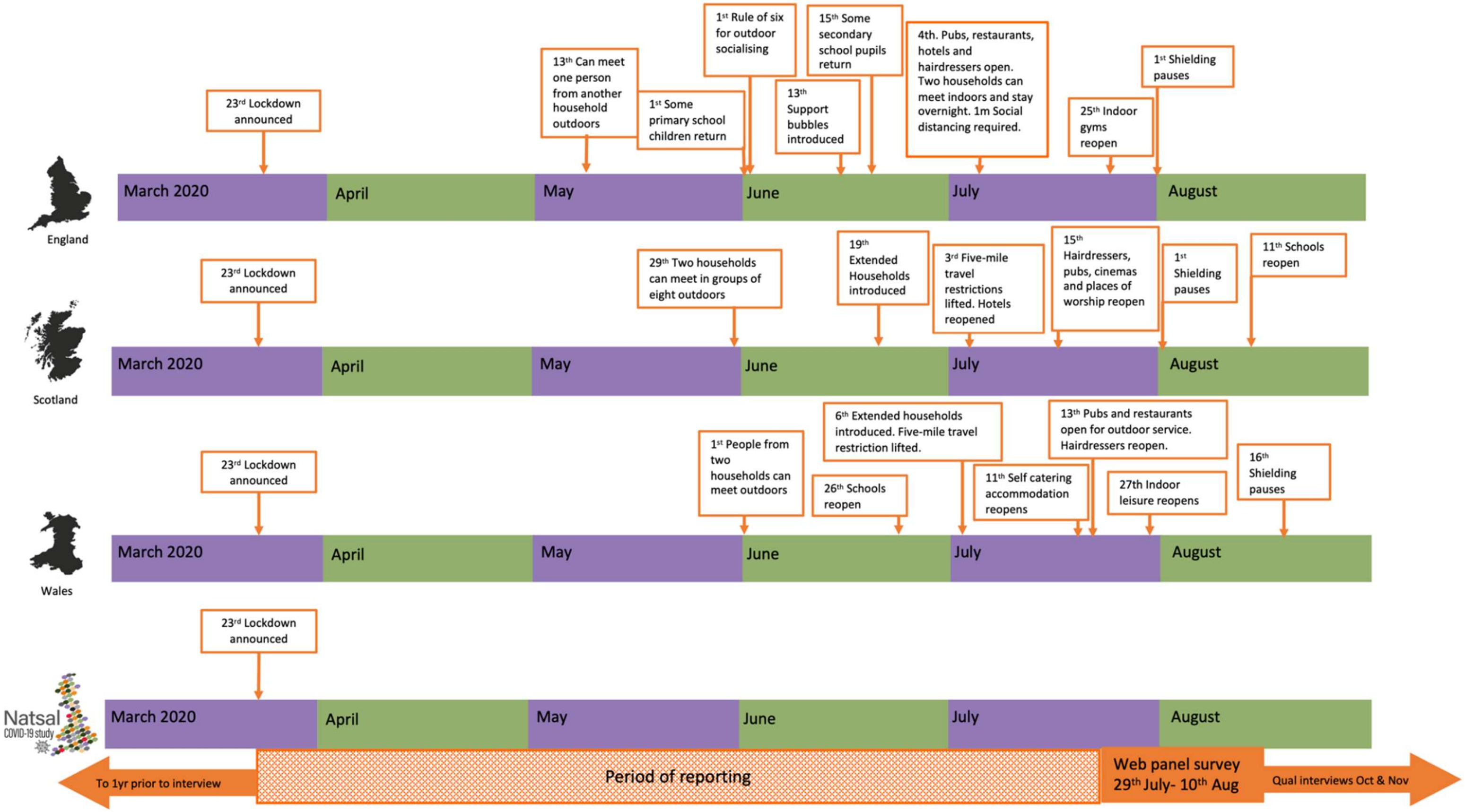

Created by the authors by adapting a figure created by Dr Caisey Pulford, Senior Surveillance and Prevention Scientist at Public Health England, who provided permission for the authors to do so. All others figures and tables created by the authors.

Appendix 2: Flowchart of the sample distribution according to participants' experience of partnered sex and relationship status since lockdown

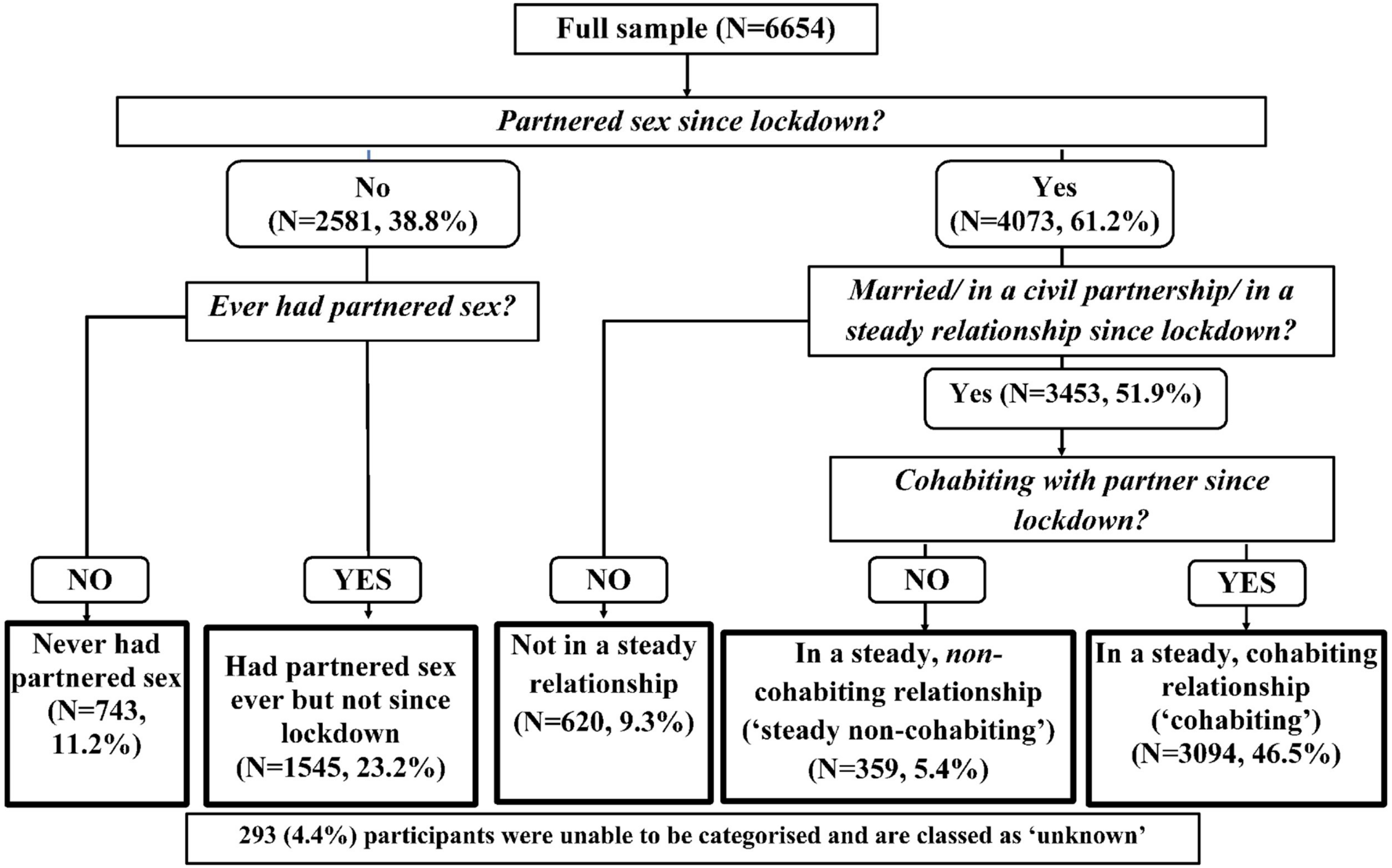

N=unweighted count. %= unweighted percentage

Appendix 3a: Experience of partnered sex by relationship status since lockdown stratified by age-group: Men ∞

		Age-group					
		**18-24**	**25-34**	**35-44**	**45-59**	**All**	**p-value**
**No partnered sex since lockdown**	**No partner sex ever % (CI)**	27·9 (23·2 - 33·1)	6·9 (5·1 - 9·2)	7·6 (5·7 - 10·1)	9·7 (8·1 - 11·6)	11·0 (9·8 - 12·3)	<0·001
	**Partnered sex ever but not since lockdown % (CI)**	13·0 (9·8 - 17·1)	18·1 (15·3 - 21·3)	21·4 (18·3 - 24·8)	36·9 (34·1 - 39·8)	25·1 (23·5 - 26·7)	
**Had partnered sex since lockdown**	**Not in a steady relationship % (CI)**	24·7 (20·3 - 29·7)	14·7 (12·2 - 17·6)	8·5 (6·6 - 11·0)	5·3 (4·1 - 6·8)	11·2 (10·0 - 12·4)	
	**In a steady non-cohabiting relationship % (CI)**	9·1 (6·5 - 12·7)	5·5 (4·1 - 7·5)	3·1 (2·0 - 4·7)	2·8 (2·0 - 4·0)	4·5 (3·7 - 5·3)	
	**In a steady cohabiting relationship % (CI)**	25·3 (20·8 - 30·3)	54·9 (51·0 - 58·6)	59·4 (55·5 - 63·2)	45·3 (42·4 - 48·2)	48·4 (46·5 - 50·2)	
**All**		100·0	100·0	100·0	100·0	100·0	
Denominators (unweighted, weighted) ǂ		*401, 450*	*775, 844*	*737, 789*	*1275, 1226*	*3187, 3310*	

Appendix 3b: Experience of partnered sex by relationship status since lockdown stratified by age-group: Women ∞

		Age-group					
		**18-24**	**25-34**	**35-44**	**45-59**	**All**	**p-value**
**No partnered sex since lockdown**	**No partner sex ever % (CI)**	26·8 (23·0 - 31·1)	10·0 (8·2 - 12·3)	10·9 (8·6 - 13·6)	11·8 (9·8 - 14·2)	12·9 (11·7 - 14·2)	<0·001
	**Partnered sex ever but not since lockdown % (CI)**	19·4 (16·1 - 23·2)	14·0 (12·2 - 35·0)	21·6 (18·6 - 24·9)	36·8 (33·6 - 40·1)	24·7 (23·1 - 26·3)	
**Had partnered sex since lockdown**	**Not in a steady relationship % (CI)**	15·6 (12·6 - 19·3)	9·9 (8·1 - 12·1)	5·1 (3·7 - 7·1)	2·8 (1·9 - 4·2)	6·9 (6·1 - 7·8)	
	**In a steady non-cohabiting relationship % (CI)**	13·6 (10·9 - 16·8)	7·7 (6·1 - 9·5)	3·6 (2·4 - 5·4)	3·3 (2·3 - 4·7)	5·8 (5·0 - 6·7)	
	**In a steady cohabiting relationship % (CI)**	24·6 (21·0 - 28·5)	58·3 (55·1 - 61·5)	58·9 (55·0 - 62·6)	45·3 (42·0 - 48·6)	49·7 (47·9 - 51·6)	
**All**		100·0	100·0	100·0	100·0	100·0	
Denominators (unweighted, weighted) ǂ		*596, 399*	*1147, 917*	*735, 813*	*965, 1190*	*3443, 3320*	

CI=confidence intervals.
ǂ Denominator: All men/women (including trans men/women)
∞ 24 participants who identified "in another way" are included in data presented for all participants but excluded from "Men" and "Women". Trans men and trans women are included in data for men and women, respectively.

Appendix 4a: Type and frequency of sexual activity reported since lockdown stratified by experience of partnered sex and relationship status since lockdown: Men

**Reported partnered sex since lockdown**	**No**		**Yes**				
**Sexual activity since lockdown**	**Among those reporting never having had partnered sex % (CI)**	**Among those reporting partnered sex ever but not since lockdown % (CI)**	**Among those not in a steady relationship % (CI)**	**Among those in a steady non-cohabiting relationship % (CI)**	**Among those in a steady cohabiting relationship % (CI)**	**All**	**p-value**
**% reporting any sexual activity***	72·5 (66·6 - 77·6)	80·3 (77·0 - 83·1)	100	100	100	92·4 (91·3 - 93·4)	<0·0001
Denominator (unwgt, wgt)	*286, 308*	*750, 765*	*359, 356*	*138, 142*	*1476, 1541*	*3009, 3112*	
**Physical sexual activities since lockdown†**							
**% reporting any physical sexual activity***	61·3 (55·1 - 67·1)	74·7 (71·2 - 77·9)	100	100	100	90 (88·8 - 91·1)	<0·0001
Denominator (unwgt, wgt)	*286, 309*	*733, 744*	*359, 356*	*138, 142*	*1476, 1541*	*2992, 3093*	
**% reporting any vaginal, oral, and/or anal sex***	0	0	90·4 (86·4 - 93·4)	96·6 (90·1 - 98·9)	98·3 (97·5 - 98·9)	62·3 (60·4 - 64·1)	<0·0001
Denominator (unwgt, wgt)	*301, 331*	*782, 799*	*357, 354*	*138, 142*	*1473, 1538*	*3051, 3163,*	
Of those who did, frequency since lockdown**:							
Less than weekly	··	··	48·6 (42·6 - 54·6)	50·2 (41·3 - 59·0)	41·5 (38·8 - 44·2)	43·3 (40·9 - 45·6)	0·030
At least once a week	··	··	51·5 (45·4 - 57·4)	49·8 (41·0 - 58·7)	58·5 (55·8 - 61·2)	56·8 (54·4 - 59·1)	
Denominator (unwgt, wgt)	··	··	*325, 320*	*134, 137*	*1443, 1512*	*1902, 1969*	
**% reporting other contact with someone's genital area***	0	0	90·2 (85·9 - 93·3)	86·3 (79·3 - 91·2)	87·3 (85·4 - 89·0)	56·2 (54·3 - 58·1)	0·36
Denominator (unwgt, wgt)	*303, 332*	*782, 799*	*349, 346*	*138, 142*	*1459, 1524*	*3031, 3142*	
Of those who did, frequency since lockdown**:							
Less than weekly	··	··	52·2 (46·1 - 58·2)	45·9 (36·6 - 55·5)	36·9 (34·2 - 39·8)	40·2 (37·8 - 42·8)	<0·0001
At least once a week	··	··	47·8 (41·8 - 53·9)	54·1 (44·6 - 63·4)	63·1 (60·2 - 65·8)	59·8 (57·3 - 62·2)	
Denominator (unwgt, wgt)	··	··	*318, 312*	*119, 123*	*1280, 1331*	*1717, 1765*	
**% reporting masturbation***	59·4 (53·3 - 65·3)	74·4 (70·8 - 77·6)	84·5 (79·6 - 88·4)	78·7 (70·7 - 85·0)	71·4 (68·8 - 73·8)	72·7 (70·9 – 74·4)	<0·0001
Denominator (unwgt, wgt)	*292, 316*	*734, 746*	*347, 342*	*136, 140*	*1433, 1495*	*2942, 3039*	
Of those who did, frequency since lockdown**:							
Less than weekly	23·5 (17·1 - 31·2)	15·9 (13·0 - 19·4)	22·1 (17·2 - 27·8)	27·5 (19·3 - 37·7)	27·0 (24·2 - 29·9)	23·3 (21·4 - 25·3)	<0·0001
At least once a week	76·6 (68·8,82·9)	84·1 (80·6,87·0)	78·0 (72·2,82·8)	72·5 (62·3,80·7)	73·0 (70·1,75·8)	76·7 (74·7,78·6)	
Denominator (unwgt, wgt)	*179, 188*	*557, 555*	*302, 289*	*108, 110*	*1047, 1068*	*2193, 2209*	
**% reporting using sex toys (by yourself or with someone else)***	3·2 (1·8 - 5·5)	5·5 (4·0 - 7·5)	46·1 (40·3 - 51·9)	33·8 (25·9 - 42·7)	29·9 (27·4 - 32·4)	22·8 (21·3 - 24·4)	<0·0001
Denominator (unwgt, wgt)	*306, 335*	*776, 792*	*348, 344*	*137, 141*	*1457, 1523*	*3024, 3134*	
Of those who did, frequency since lockdown**:							
Less than weekly	··	54·4 (38·3 - 69·6)	41·4 (33·4 - 50·0)	57·0 (41·4 - 71·3)	49·1 (44·1 - 54·1)	47·9 (44·0 - 51·9)	0·21
At least once a week	··	45·6 (30·4 - 61·7)	58·6 (50·0 - 66·6)	43·0 (28·7 - 58·6)	50·9 (46·0 - 55·9)	52·1 (48·1 - 56·0)	
Denominator (unwgt, wgt)	*16, 11‡‡*	*51, 43*	*167, 158*	*48,48***	*451, 455*	*733, 715*	
**Virtual sexual activities since lockdown**							

**% reporting any virtual sexual activity† ***	58·5 (52·3 - 64·5)	71·6 (68·1 - 74·9)	91·9 (88·0 - 94·6)	75·4 (67·1 - 82·1)	67·8 (65·2 - 70·3)	70·9 (69·1 - 72·6)	<0·0001
Denominator (unwgt, wgt)	*287, 311,*	*759, 774*	*353, 349*	*137, 141*	*1448, 1513*	*2984, 3088*	
**% reporting virtual sexual activity excluding looking at pornography†† ***	19·0 (14·6 - 24·4)	27·8 (24·5 - 31·3)	80·4 (75·4 - 84·6)	58·6 (49·8 - 66·9)	27·6 (25·2 - 30·1)	34·1 (32·3 - 35·9)	<0·0001
Denominator (unwgt, wgt)	*297, 324*	*776, 792*	*354, 351*	*137, 141*	*1466, 1531*	*3030, 3138*	
**% reporting messaging via dating apps/online***	16·2 (12·1 - 21·3)	24·1 (21·0 - 27·5)	66·9 (61·2 - 72·1)	40·2 (31·9 - 49·2)	19·2 (17·1 - 21·5)	26·4 (24·7 - 28·1)	<0·0001
Denominator (unwgt, wgt)	*304, 331*	*779, 795*	*351, 348*	*137, 141*	*1459, 1526*	*3030, 3141*	
Of those who did, frequency since lockdown**:							
Less than weekly	57·4 (42·0 - 71·5)	44·2 (36·6 - 52·1)	27·2 (21·4 - 34·0)	20·3 (11·1 - 34·3)	27·9 (22·5 - 33·9)	32·9 (29·4 - 36·5)	<0·0001
At least once a week	42·6 (28·5 - 58·0)	55·8 (47·9 - 63·4)	72·8 (66·0 - 78·6)	79·7 (65·8 - 88·9)	72·1 (66·1 - 77·5)	67·1 (63·5 - 70·6)	
Denominator (unwgt, wgt)	*50, 54*	*188, 192*	*244, 233*	*53, 57*	*290, 293*	*825, 828*	
**% reporting sexting (images or recorded videos)***	4·5 (2·6 - 7·8)	10·6 (8·5 - 13·1)	56·9 (51·1 - 62·5)	40·1 (31·7 - 49·1)	20·7 (18·6 - 23·0)	21·4 (19·8 - 23·0)	<0·0001
Denominator (unwgt, wgt)	*303, 331*	*780, 796*	*355, 352*	*137, 142*	*1468, 1535*	*3155, 3043*	
Of those who did, frequency since lockdown**:							
Less than weekly	··	51·2 (39·9 - 62·4)	45·0 (37·5 - 52·7)	61·7 (47·1 - 74·5)	42·7 (36·9 - 48·7)	46·4 (42·3 - 50·6)	0·10
At least once a week	··	48·8 (37·6 - 60·1)	55·1 (47·4 - 62·5)	38·3 (25·5 - 52·9)	57·3 (51·3 - 63·1)	53·6 (49·4 - 57·7)	
Denominator (unwgt, wgt)	*6, 15 ‡*	*93, 84*	*207, 200*	*53, 57*	*314, 318*	*683, 674*	
**% reporting using video or voice calls to interact with someone sexually***	3·3 (1·6 - 6·7)	6·8 (5·1 - 9·0)	53·8 (48·1 - 59·5)	39·5 (31·2 - 48·5)	16·1 (14·2 - 18·2)	17·6 (16·2 - 19·2)	<0·0001
Denominator (unwgt, wgt)	*305, 334*	*778, 794*	*353, 349*	*138, 142*	*1465, 1531*	*3039, 3150*	
Of those who did, frequency since lockdown**							
Less than weekly	··	50·8 (36·4 - 65·2)	43·2 (35·5 - 51·2)	48·5 (34·5 - 62·8)	43·0 (36·5 - 49·8)	45·1 (40·6 - 49·7)	0·21
At least once a week	··	49·2 (34·8 - 63·6)	56·8 (48·8 - 64·5)	51·5 (37·2 - 65·6)	57·0 (50·2 - 63·6)	54·9 (50·3 - 59·4)	
Denominator (unwgt, wgt)	*9, 11 ‡*	*50, 54*	*186, 188,*	*51, 56*	*237, 247*	*533, 555*	
**% reporting looking at pornography***	50·1 (43·9 - 56·2)	67·1 (63·5 - 70·6)	81·2 (76·2 - 85·4)	67·5 (58·8 - 75·2)	63·4 (60·7 - 66·0)	65·1 (63·3 - 66·9)	<0·0001
Denominator (unwgt, wgt)	*294, 319*	*750, 763*	*348, 345*	*136, 140*	*1445, 1510*	*2973, 3076*	
Of those who did, frequency since lockdown**:							
Less than weekly	29·5 (22·0 - 38·2)	22·0 (18·4 - 26·0)	32·6 (26·8 - 39·1)	24·1 (16·1 - 34·5)	33·3 (30·1 - 36·6)	29·6 (27·4 - 31·8)	<0·0001
At least once a week	70·5 (61·8 - 78·0)	78·0 (74·0 - 81·6)	67·4 (60·9 - 73·2)	75·9 (65·6 - 83·9)	66·7 (63·4 - 69·9)	70·4 (68·2 - 72·6)	
Denominator (unwgt, wgt)	*151, 160*	*511, 512*	*286, 281*	*93, 94*	*938, 957*	*1979, 2003*	
**% reporting paying for online sexual services (e.g. live streaming)***	1·3 (0·5 - 3·4)	1·1 (0·5 - 2·2)	32·7 (27·4 - 38·4)	16·4 (10·7 - 24·4)	11·2 (9·6 - 13·1)	10·2 (9·1 - 11·4)	<0·0001
Denominator (unwgt, wgt)	*309, 338*	*780, 796*	*352, 350*	*137, 141*	*1466, 1531*	*3044, 3155*	
Of those who did, frequency since lockdown**:							
Less than weekly	··	··	47·2 (37·0 - 57·6)	··	40·0 (32·2 - 48·2)	42·9 (37·0 - 49·0)	0·11
At least once a week	··	··	52·8 (42·4 - 63·0)	··	60·0 (51·8 - 67·8)	57·1 (51·0 - 63·0)	
Denominator (unwgt, wgt)	*5, 5 ‡*	*8, 8 ‡*	*106, 114*	*22, 23 ‡*	*163, 172*	*304, 322*	

Mercer CH, *et al. Sex Transm Infect* 2021;0:1–9. doi: 10.1136/sextrans-2021-055210

CI=confidence intervals·
* Denominator: All respondents
** Denominator: All respondents who reported the activity in lockdown
† Reported at least one of the following since lockdown: vaginal, anal or oral sex, other contact with someone's genital area, masturbating, using sex toys (by yourself or with someone else)
†† Reported at least one of the following since lockdown: messaging via dating apps/online, sexting (images or recorded videos), using video or voice calls to interact with someone sexually, looking at pornography, paying for online sexual services (e.g. live streaming)
††† Reported at least one of the following since lockdown: messaging via dating apps/online, sexting (images or recorded videos), using video or voice calls to interact with someone sexually, paying for online sexual services (e.g. live streaming)
ǂ Unweighted denominator <30· Results not shown due to small denominator·

Mercer CH, *et al. Sex Transm Infect* 2021;0:1–9. doi: 10.1136/sextrans-2021-055210

Appendix 4b: Type and frequency of sexual activity reported since lockdown stratified by experience of partnered sex and relationship status since lockdown: Women∞

Reported partnered sex since lockdown	No		Yes				
**Sexual activity since lockdown**	**Among those reporting never having had partnered sex % (CI)**	**Among those reporting partnered sex ever but not since lockdown % (CI)**	**Among those not in a steady relationship % (CI)**	**Among those in a steady non-cohabiting relationship % (CI)**	**Among those in a steady cohabiting relationship % (CI)**	**All**	**p-value**
**% reporting any sexual activity***	36·1 (30·8 - 41·7)	50·0 (46·1 - 53·8)	100	100	100	80·7 (79·1 - 82·1)	<0·0001
Denominator (unwgt, wgt)	*346, 333*	*732, 748*	*258, 217*	*220, 182*	*1612, 1564*	*3168, 3044*	
**% reporting physical sexual activity †***	24·5 (20·0 - 29·6)	41·5 (37·7 - 45·3)	100	100	100	77·4 (75·7 - 78·9)	<0·0001
Denominator (unwgt, wgt)	*349, 339*	*720, 740*	*258, 217*	*220, 182*	*1612, 1564*	*3159, 3041*	
**% reporting vaginal, oral, and/or anal sex***	0	0	94·2 (89·6 - 96·8)	98·0 (94·6 - 99·3)	97·1 (96·0 - 97·9)	61·2 (59·4 - 63·0)	0·065
Denominator (unwgt, wgt)	*379, 366*	*758, 776*	*258, 217*	*219, 182*	*1609, 1562*	*3223, 3102*	
Of those who did, frequency since lockdown**:							
Less than weekly	··	··	60·4 (53·5 - 66·9)	45·0 (37·8 - 52·3)	40·0 (37·4 - 42·6)	42·6 (40·3 - 45·0)	<0·0001
At least once a week	··	··	39·6 (33·1 - 46·5)	55·1 (47·7 - 62·2)	60·0 (57·4 - 62·6)	57·4 (55·1 - 59·7)	
Denominator (unwgt, wgt)	··	··	*245, 204*	*214, 178*	*1568, 1516*	*2027, 1898*	
**% reporting other contact with someone's genital area***	0	0	80·9 (75·0 - 85·7)	85·6 (79·1 - 90·3)	83·8 (81·8 - 85·7)	52·4 (50·5 - 54·3)	0·460
Denominator (unwgt, wgt)	*380, 369*	*758, 776*	*251, 210*	*218, 181*	*1576, 1525*	*3183, 3060*	
Of those who did, frequency since lockdown**:							
Less than weekly	··	··	61·1 (53·6 - 68·2)	41·1 (33·9 - 48·8)	36·1 (33·4 - 38·9)	39·2 (36·8 - 41·7)	<0·0001
At least once a week	··	··	38·9 (31·8 - 46·4)	58·9 (51·2 - 66·2)	63·9 (61·1 - 66·6)	60·8 (58·3 - 63·2)	
Denominator (unwgt, wgt)	··	··	*207, 170*	*192, 155*	*1344, 1279*	*1743, 1603*	
**% reporting masturbation***	21·5 (17·5 - 26·2)	39·1 (35·4 - 42·9)	65·3 (58·4 - 71·7)	60·7 (53·2 - 67·8)	42·9 (40·3 - 45·5)	41·8 (40·0 - 43·7)	<0·0001
Denominator (unwgt, wgt)	*374, 369*	*721, 740*	*245, 203*	*206, 169*	*1570, 1526*	*3116, 3007*	
Of those who did, frequency since lockdown**:							
Less than weekly	43·3 (32·5 - 54·8)	54·4 (48·5 - 60·2)	40·0 (32·3 - 48·3)	53·5 (44·1 - 62·7)	52·6 (48·7 - 56·5)	51·2 (48·4 - 54·0)	0·034
At least once a week	56·7 (45·2,67·5)	45·6 (39·8,51·6)	60·0 (51·7,67·7)	46·5 (37·4,55·9)	47·4 (43·5,51·3)	48·8 (46·0,51·6)	
Denominator (unwgt, wgt)	*95, 79*	*321, 289*	*174, 133*	*130, 103*	*737, 654,*	*1457, 1257*	
**% reporting using sex toys (by yourself or with someone else)***	5·9 (3·9 - 8·9)	18·2 (15·6 - 21·2)	48·6 (41·9 - 55·3)	40·7 (33·7 - 48·1)	27·0 (24·8 - 29·3)	24·4 (22·9 - 26·0)	<0·0001
Denominator (unwgt, wgt)	*391, 382*	*737, 756*	*250, 207*	*211, 173*	*1587, 1543*	*3176, 3062*	
Of those who did, frequency since lockdown**:							
Less than weekly	··	53·9 (45·5 - 62·1)	41·8 (32·9 - 51·4)	63·5 (52·0 - 73·6)	58·7 (53·8 - 63·4)	55·8 (52·2 - 59·4)	0·023
At least once a week	··	46·1 (37·9 - 54·5)	58·2 (48·6 - 67·2)	36·5 (26·4 - 48·0)	41·3 (36·7 - 46·2)	44·2 (40·6 - 47·8)	
Denominator (unwgt, wgt)	*24, 23 ‡‡*	*160, 138*	*132, 101*	*90, 71*	*488, 416*	*894, 748*	
**Any virtual sexual activity since lockdown**							
**% reporting any virtual sexual activity†† ***	25·7 (21·3 - 30·6)	29·7 (26·5 - 33·1)	71·5 (64·9 - 77·3)	62·9 (55·5 - 69·6)	29·3 (27·0 - 31·7)	33·9 (32·2 - 35·6)	<0·0001
Denominator (unwgt, wgt)	*380, 372*	*743, 761*	*254, 213*	*218, 181*	*1591, 1545*	*3186, 3070*	
**% reporting any virtual sexual activity excluding looking at pornography ††† ***	19·5 (15·7 - 23·9)	23·2 (20·3 - 26·4)	62·4 (55·6 - 68·7)	51·0 (43·7 - 58·2)	14·3 (12·7 - 16·2)	22·7 (21·2 - 24·2)	<0·0001
Denominator (unwgt, wgt)	*383, 374*	*746, 764*	*256, 215*	*218, 181*	*1596, 1549*	*3199, 3082*	

**% reporting messaging via dating apps/online***	15·7 (12·4 - 19·8)	19·1 (16·5 - 22·1)	47·4 (40·8 - 54·1)	36·6 (29·9 - 43·8)	8·3 (7·0 - 9·9)	16·3 (15·0 - 17·6)	<0·0001
Denominator (unwgt, wgt)	*391, 381*	*753, 771*	*254, 213*	*219, 182*	*1601, 1553*	*3218, 3099*	
Of those who did, frequency since lockdown**:							
Less than weekly	52·3 (39·9 - 64·5)	36·7 (29·4 - 44·7)	30·0 (21·7 - 39·8)	19·3 (10·6 - 32·7)	28·3 (20·8 - 37·2)	32·7 (28·7 - 37·1)	0·002
At least once a week	47·7 (35·5 - 60·1)	63·3 (55·3 - 70·6)	70·1 (60·3 - 78·3)	80·7 (67·3 - 89·4)	71·8 (62·8 - 79·2)	67·3 (62·9 - 71·3)	
Denominator (unwgt, wgt)	*75, 60*	*178, 147*	*127, 101*	*86, 66*	*151, 130*	*617, 504*	
**% reporting sexting (images or recorded videos)***	4·9 (3·2 - 7·6)	8·5 (6·7 - 10·8)	37·2 (31·0 - 43·8)	32·5 (26·2 - 39·4)	9·7 (8·3 - 11·3)	12·0 (10·9 - 13·2)	<0·0001
Denominator (unwgt, wgt)	*397, 389*	*747, 766*	*253, 213*	*219, 182*	*1606, 1558*	*3222, 3106*	
Of those who did, frequency since lockdown**:							
Less than weekly	··	50·5 (38·5 - 62·5)	44·4 (34·1 - 55·3)	50·0 (38·1 - 61·9)	51·9 (44·0 - 59·8)	50·8 (45·8 - 55·8)	0·30
At least once a week	··	49·5 (37·6 - 61·5)	55·6 (44·8 - 65·9)	50·0 (38·1 - 61·9)	48·1 (40·2 - 56·1)	49·2 (44·2 - 54·2)	
Denominator (unwgt, wgt)	*24, 19 ǂǂ*	*75, 65,*	*105, 79*	*77, 59*	*185, 151*	*466, 373*	
**% reporting using video or voice calls to interact with someone sexually***	3·0 (1·6 - 5·4)	4·9 (3·6 - 6·7)	28·1 (22·4 - 34·7)	24·2 (18·7 - 30·8)	6·9 (5·7 - 8·3)	8·4 (7·4 - 9·4)	<0·0001
Denominator (unwgt, wgt)	*397, 387*	*749, 768*	*256, 214*	*217, 180*	*1605, 1557*	*3224, 3106*	
Of those who did, frequency since lockdown**:							
Less than weekly	··	59·8 (43·5 - 74·2)	51·9 (38·9 - 64·7)	48·0 (34·5 - 61·9)	47·2 (37·5 - 57·0)	50·8 (44·6 - 57·0)	0·69
At least once a week	··	40·2 (25·8 - 56·5)	48·1 (35·3 - 61·1)	52·0 (38·1 - 65·5)	52·8 (43·0 - 62·5)	49·2 (43·0 - 55·4)	
Denominator (unwgt, wgt)	*11, 11 ǂǂ*	*42, 38 ǂ*	*68, 60*	*56, 44*	*123, 107*	*300, 260*	
**% reporting looking at pornography***	11·6 (8·6 - 15·6)	14·7 (12·4 - 17·4)	44·8 (38·2 - 51·6)	35·2 (28·6 - 42·5)	21·9 (19·8 - 24·1)	21·1 (19·7 - 22·7)	<0·0001
Denominator (unwgt, wgt)	*388, 381*	*742, 761*	*249, 206*	*215, 180*	*1597, 1551*	*3191, 3078*	
Of those who did, frequency since lockdown**:							
Less than weekly	47·5 (32·4 - 63·1)	60·4 (51·5 - 68·7)	57·0 (47·0 - 66·4)	59·8 (47·5 - 70·9)	65·0 (59·6 - 70·0)	61·4 (57·6 - 65·0)	0·18
At least once a week	52·5 (36·9 - 67·6)	39·6 (31·3 - 48·5)	43·0 (33·6 - 53·0)	40·2 (29·1 - 52·5)	35·0 (30·0 - 40·4)	38·6 (35·0 - 42·5)	
Denominator (unwgt, wgt)	*51, 44*	*144, 112*	*124, 92*	*84, 63*	*395, 339*	*798· 651*	
**% reporting paying for online sexual services (e.g. live streaming)***	0·5 (0·1 - 2·0)	0·4 (0·1 - 1·2)	9·0 (5·8 - 13·8)	4·9 (2·7 - 8·8)	2·8 (2·1 - 3·8)	2·4 (1·9 - 3·1)	<0·0001
Denominator (unwgt, wgt)	*400, 391*	*757, 775*	*253, 210*	*219, 182*	*1607, 1560*	*3236, 3117*	
Of those who did, frequency since lockdown**:							
Less than weekly	··	··	··	··	41·3 (27·4 - 56·8)	41·3 (30·7 - 52·7)	0·26
At least once a week	··	··	··	··	58·7 (43·2 - 72·6)	58·7 (47·3 - 69·3)	
Denominator (unwgt, wgt)	*2, 2ǂǂ*	*4, 3 ǂǂ*	*24, 19 ǂǂ*	*12, 9 ǂǂ*	*53, 43*	*95, 76*	

CI=confidence intervals.
∞ 24 participants who identified "in another way" are included in data presented for all participants but excluded from "Men" and "Women". Trans men and trans women are included in data for men and women, respectively.
* Denominator: All respondents
** Denominator: All respondents who reported the activity in lockdown
† Reported at least one of the following since lockdown: vaginal, anal or oral sex, other contact with someone's genital area, masturbating, using sex toys (by yourself or with someone else)
†† Reported at least one of the following since lockdown: messaging via dating apps/online, sexting (images or recorded videos), using video or voice calls to interact with someone sexually, looking at pornography, paying for online sexual services (e.g. live streaming)
††† Reported at least one of the following since lockdown: messaging via dating apps/online, sexting (images or recorded videos), using video or voice calls to interact with someone sexually, paying for online sexual services (e.g. live streaming)
ǂ Unweighted denominator <50. Results should be interpreted with caution due to small denominator.
ǂǂ Unweighted denominator <30. Results not shown due to small denominator.

Mercer CH, *et al. Sex Transm Infect* 2021;0:1–9. doi: 10.1136/sextrans-2021-055210

Appendix 5a: Type and frequency of sexual activity reported since lockdown stratified by experience of partnered sex and relationship status since lockdown: Age-group 18-24 years

Reported partnered sex since lockdown	No		Yes			All	p-value
Sexual activity since lockdown	Among those reporting never having had partnered sex % (CI)	Among those reporting partnered sex ever but not since lockdown % (CI)	Among those not in a steady relationship % (CI)	Among those in a steady non-cohabiting relationship % (CI)	Among those in a steady cohabiting relationship % (CI)		
**% reporting any sexual activity***	62·5 (55·2 - 69·2)	85·5 (77·4 - 91·0)	100	100	100	88·4 (85·8 - 90·5)	<0·0001
Denominator (unwgt, wgt)	*215, 195*	*159, 128*	*187, 171*	*115, 91*	*252, 205*	*928, 789*	
**% reporting any physical sexual activity † ***	46·3 (39·1 - 53·8)	68·2 (59·2 - 76·0)	100	100	100	81·5 (78·6 - 84·2)	<0·0001
Denominator (unwgt, wgt)	*216, 199*	*152, 122*	*187, 171*	*115, 91*	*252, 205*	*922, 787*	
**% reporting any virtual sexual activity †† ***	51·3 (44·1 - 58·5)	80·1 (71·7 - 86·5)	91·0 (84·6 - 95·0)	79·6 (70·8 - 86·2)	68·9 (62·2 - 74·9)	72·2 (68·9 - 75·3)	<0·0001
Denominator (unwgt, wgt)	*225, 202*	*160, 129*	*185, 169*	*115, 91*	*248, 202*	*933, 793*	
**% reporting any virtual sexual activity excluding looking at pornography ††† ***	25·6 (19·9 - 32·3)	61·3 (52·4 - 69·5)	86·0 (79·2 - 90·9)	69·4 (59·7 - 77·6)	47·6 (40·6 - 54·7)	54·6 (51·0 - 58·2)	<0·0001
Denominator (unwgt, wgt)	*231, 211*	*160, 129*	*185, 169*	*115, 91*	*251, 204*	*942, 804*	

CI=confidence intervals.
* Denominator: All respondents
† Reported at least one of the following since lockdown: vaginal, anal or oral sex, other contact with someone's genital area, masturbating, using sex toys (by yourself or with someone else)
†† Reported at least one of the following since lockdown: messaging via dating apps/online, sexting (images or recorded videos), using video or voice calls to interact with someone sexually, looking at pornography, paying for online sexual services (e.g. live streaming)
††† Reported at least one of the following since lockdown: messaging via dating apps/online, sexting (images or recorded videos), using video or voice calls to interact with someone sexually, paying for online sexual services (e.g. live streaming)

Mercer CH, *et al. Sex Transm Infect* 2021;0:1–9. doi: 10.1136/sextrans-2021-055210

Appendix 5b: Type and frequency of sexual activity reported since lockdown stratified by experience of partnered sex and relationship status since lockdown: Age-group 25-34 years

**Reported partnered sex since lockdown**	**No**		**Yes**				
**Sexual activity since lockdown**	**Among those reporting never having had partnered sex % (CI)**	**Among those reporting partnered sex ever but not since lockdown % (CI)**	**Among those not in a steady relationship % (CI)**	**Among those in a steady non-cohabiting relationship % (CI)**	**Among those in a steady cohabiting relationship % (CI)**	**All**	**p-value**
**% reporting any sexual activity***	53·5 (44·0 - 62·7)	81·9 (76·5 - 86·2)	100	100	100	93·6 (92·3 - 94·8)	<0·0001
Denominator (unwgt, wgt)	*130, 126*	*282, 261*	*229, 207*	*128, 112*	*1049, 959*	*1818, 1664*	
**% reporting any physical sexual activity † ***	40·1 (31·1 - 49·7)	72·8 (66·7 - 78·1)	100	100	100	91·3 (89·7 - 92·6)	<0·0001
Denominator (unwgt, wgt)	*132, 129*	*270, 248*	*229, 207*	*128, 112*	*1049, 959*	*1808, 1654*	
**% reporting virtual sexual activity †† ***	47·2 (38·1 - 56·6)	71·8 (65·9 - 77·1)	86·7 (81·3 - 90·7)	69·4 (60·1 - 77·4)	56·1 (52·8 - 59·3)	62·6 (60·1 - 65·0)	<0·0001
Denominator (unwgt, wgt)	*134, 131*	*286, 265*	*226, 204*	*126, 110*	*1026, 937*	*1798, 1646*	
**% reporting any virtual sexual activity excluding looking at pornography ††† ***	24·5 (17·7 - 33·0)	49·1 (42·9 - 55·4)	76·0 (69·4 - 81·6)	60·3 (50·9 - 69·1)	28·1 (25·2 - 31·2)	39·2 (36·8 - 41·7)	<0·0001
Denominator (unwgt, wgt)	*138, 134*	*290, 269*	*227, 205*	*126, 110*	*1036, 946*	*1817, 1664*	

CI=confidence intervals.
* Denominator: All respondents
† Reported at least one of the following since lockdown: vaginal, anal or oral sex, other contact with someone's genital area, masturbating, using sex toys (by yourself or with someone else)
†† Reported at least one of the following since lockdown: messaging via dating apps/online, sexting (images or recorded videos), using video or voice calls to interact with someone sexually, looking at pornography, paying for online sexual services (e.g. live streaming)
††† Reported at least one of the following since lockdown: messaging via dating apps/online, sexting (images or recorded videos), using video or voice calls to interact with someone sexually, paying for online sexual services (e.g. live streaming)

Appendix 5c: Type and frequency of sexual activity reported since lockdown stratified by experience of partnered sex and relationship status since lockdown: Age-group 35-44 years

Reported partnered sex since lockdown	No		Yes				
**Sexual activity since lockdown**	**Among those reporting never having had partnered sex % (CI)**	**Among those reporting partnered sex ever but not since lockdown % (CI)**	**Among those not in a steady relationship % (CI)**	**Among those in a steady non-cohabiting relationship % (CI)**	**Among those in a steady cohabiting relationship % (CI)**	**All**	**p-value**
**% reporting any sexual activity***	51·9 (41·6 - 62·1)	68·2 (62·3 - 73·5)	100	100	100	89·7 (87·8 - 91·3)	<0·0001
Denominator (unwgt, wgt)	*110, 120,*	*303, 320*	*104, 104*	*49, 52 ǂ*	*825, 909*	*1390, 1505*	
**% reporting any physical sexual activity † ***	42·4 (32·6 - 52·8)	63·7 (57·6 - 69·3)	100	100	100	88·2 (86·2 - 89·9)	<0·0001
Denominator (unwgt, wgt)	*100, 109*	*294, 311*	*104, 104*	*49, 52 ǂ*	*825, 909*	*1372, 1485*	
**% reporting virtual sexual activity †† ***	34·0 (25·4 - 43·7)	56·3 (50·3 - 62·0)	83·7 (74·4 - 90·1)	67·6 (52·76 - 79·58)	50·2 (46·6 - 53·8)	52·9 (50·1 - 55·7)	<0·0001
Denominator (unwgt, wgt)	*111, 127*	*307, 325*	*100, 99*	*48, 51 ǂ*	*810, 893*	*1376, 1495*	
**% reporting any virtual sexual activity excluding looking at pornography ††† ***	19·1 (12·7 - 27·8)	28·6 (23·5 - 34·2)	72·2 (61·9 - 80·7)	43·2 (29·5 - 58·0)	20·2 (17·5 - 23·2)	26·2 (23·8 - 28·7)	<0·0001
Denominator (unwgt, wgt)	*115, 131*	*309, 327*	*101, 101*	*48, 51 ǂ*	*817, 901*	*1390, 1512*	

CI=confidence intervals.
* Denominator: All respondents
† Reported at least one of the following since lockdown: vaginal, anal or oral sex, other contact with someone's genital area, masturbating, using sex toys (by yourself or with someone else)
†† Reported at least one of the following since lockdown: messaging via dating apps/online, sexting (images or recorded videos), using video or voice calls to interact with someone sexually, looking at pornography, paying for online sexual services (e.g. live streaming)
††† Reported at least one of the following since lockdown: messaging via dating apps/online, sexting (images or recorded videos), using video or voice calls to interact with someone sexually, paying for online sexual services (e.g. live streaming)
ǂ Unweighted denominator <50. Results should be interpreted with caution due to small denominator.

Mercer CH, *et al. Sex Transm Infect* 2021;0:1–9. doi: 10.1136/sextrans-2021-055210

Appendix 5d: Type and frequency of sexual activity reported since lockdown stratified by experience of partnered sex and relationship status since lockdown: Age-group 45-59 years

**Reported partnered sex since lockdown**	**No**		**Yes**				
**Sexual activity since lockdown**	**Among those reporting never having had partnered sex % (CI)**	**Among those reporting partnered sex ever but not since lockdown % (CI)**	**Among those not in a steady relationship % (CI)**	**Among those in a steady non-cohabiting relationship % (CI)**	**Among those in a steady cohabiting relationship % (CI)**	**All**	**p-value**
**% reporting any sexual activity***	47·0 (39·8 - 54·5)	55·8 (52·1 - 59·5)	100	100	100	78·8 (76·9 - 80·6)	<0·0001
Denominator (unwgt, wgt)	*193, 218*	*744, 809*	*100, 94*	*67, 70*	*968, 1039*	*2072, 2230*	
**% reporting any physical sexual activity † ***	39·1 (32·3 - 46·4)	50·3 (46·5 - 54·0)	100	100	100	76·0 (74·0 - 77·9)	<0·0001
Denominator (unwgt, wgt)	*194, 219*	*742, 808*	*100, 94*	*67, 70*	*968, 1039*	*2071, 2229*	
**% reporting any virtual sexual activity †† ***	32·1 (25·8 - 39·0)	37·6 (34·2 - 41·2)	67·3 (56·4 - 76·6)	53·0 (40·3 - 65·3)	36·0 (33·0 - 39·2)	38·0 (35·9 - 40·2)	<0·0001
Denominator (unwgt, wgt)	*204, 231*	*754, 821*	*99, 93*	*67, 70*	*961, 1031*	*2085, 2246*	
**% reporting any virtual sexual activity excluding looking at pornography ††† ***	10·8 (7·0 - 16·2)	11·3 (9·2 - 13·8)	48·0 (37·6 - 58·5)	33·9 (22·8 - 47·2)	10·0 (8·3 - 12·1)	12·9 (11·5 - 14·4)	<0·0001
Denominator (unwgt, wgt)	*203, 230*	*768, 835*	*100, 94*	*67, 70*	*964, 1035*	*2102, 2263*	

CI=confidence intervals.
* Denominator: All respondents
† Reported at least one of the following since lockdown: vaginal, anal or oral sex, other contact with someone's genital area, masturbating, using sex toys (by yourself or with someone else)
†† Reported at least one of the following since lockdown: messaging via dating apps/online, sexting (images or recorded videos), using video or voice calls to interact with someone sexually, looking at pornography, paying for online sexual services (e.g. live streaming)
††† Reported at least one of the following since lockdown: messaging via dating apps/online, sexting (images or recorded videos), using video or voice calls to interact with someone sexually, paying for online sexual services (e.g. live streaming)

Appendix 6a: Extent and direction of perceived change in frequency of particular physical and virtual sexual activities compared to pre-lockdown, stratified by experience of partnered sex and relationship status since lockdown: Men ∞

Reported partnered sex since lockdown	No		Yes				
	Among those reporting never having had partnered sex % (CI)	Among those reporting partnered sex ever but not since lockdown % (CI)	Among those not in a steady relationship % (CI)	Among those in a steady non-cohabiting relationship % (CI)	Among those in a steady cohabiting relationship % (CI)	All	p-value
**Vaginal, anal and/or oral sex**							
**Did less since lockdown**	··	100	36·9 (31·3,43·0)	47·5 (38·8,56·4)	17·3 (15·4,19·5)	31·5 (29·5,33·7)	<0·0001
**No change since lockdown**	··	0	37·0 (31·4,43·1)	38·0 (29·8,47·0)	65·0 (62·3,67·6)	51·8 (49·5,54·1)	
**Did more since lockdown**	··	0	26·0 (21·0,31·7)	14·5 (9·2,22·1)	17·7 (15·6,19·9)	16·6 (15·0,18·4)	
*Denominator (unwgt, wgt)**	··	*247, 251*	*320, 315*	*135, 137*	*1413, 1479*	*2115, 2181*	
**Other contact with someone's genital area**							
**Did less since lockdown**	··	100·0	41·6 (35·6,47·9)	42·6 (33·9,51·9)	16·3 (14·3,18·6)	31·9 (29·7,34·1)	<0·0001
**No change since lockdown**	··	0·0	35·4 (29·6,41·7)	40·0 (31·3,49·3)	65·9 (63·1,68·6)	51·8 (49·4,54·2)	
**Did more since lockdown**	··	0·0	23·0 (18·1,28·8)	17·4 (11·2,26·0)	17·8 (15·6,20·2)	16·4 (14·6,18·2)	
*Denominator (unwgt, wgt)**	··	*245, 243*	*295, 288*	*126, 130*	*1283, 1333*	*1949, 1994*	
**Masturbation**							
**Did less since lockdown**	17·9 (12·3,25·2)	12·2 (9·6,15·3)	13·7 (9·9,18·7)	11·3 (6·4,19·1)	12·4 (10·4,14·7)	12·9 (11·5,14·5)	<0·0001
**No change since lockdown**	62·0 (54·1,69·4)	67·8 (63·5,71·8)	48·3 (42·0,54·6)	49·6 (39·5,59·7)	61·2 (58·0,64·3)	60·7 (58·5,62·9)	
**Did more since lockdown**	20·1 (14·6,27·1)	20·1 (16·7,24·0)	38·0 (32·0, 44·4)	39·1 (29·6,49·6)	26·4 (23·7,29·4)	26·3 (24·4,28·4)	
*Denominator (unwgt, wgt)***	*187, 196*	*569, 565*	*294, 283*	*105, 108*	*1045, 1064*	*2200, 2216*	
**Using sex toys (by yourself or with someone else)**							
**Did less since lockdown**	70·6 (52·0,84·2)	55·9 (45·0,66·2)	26·4 (19·6,34·6)	19·6 (10·4,33·9)	19·6 (15·9,23·9)	27·0 (23·8,30·5)	<0·0001
**No change since lockdown**	23·6 (11·6,41·9)	30·8 (21·7,41·6)	46·1 (37·9,54·5)	37·7 (24·8,52·7)	55·6 (50·6,60·4)	48·5 (44·8,52·3)	
**Did more since lockdown**	5·9 (1·5,20·7)	13·4 (7·4,23·0)	27·5 (20·5,35·8)	42·7 (28·7,58·0)	24·9 (20·9,29·3)	24·5 (21·4,27·9)	
*Denominators (wgt, unwgt)***	*33, 29ǂ*	*105, 94*	*167, 162*	*50, 51*	*471, 474*	*826, 810*	
**Messaging via dating apps/ online**							
**Did less since lockdown**	44·1 (31·5,57·5)	42·0 (35·5,48·7)	23·7 (18·2, 30·2)	27·3 (17·0,40·6)	27·5 (22·4,33·2)	31·7 (28·5,35·0)	0·002
**No change since lockdown**	36·7 (25·1,50·2)	31·9 (25·9,38·5)	39·7 (33·0,46·7)	37·6 (25·5,51·5)	38·8 (33·2,44·8)	36·9 (33·6,40·4)	
**Did more since lockdown**	19·2 (10·8,31·7)	26·2 (20·6,32·6)	36·7 (30·1,43·7)	35·1 (23·4,48·9)	33·7 (28·3,39·6)	31·4 (28·3,34·8)	
*Denominators (wgt, unwgt)***	*68, 65*	*253, 249*	*232, 238*	*61, 58*	*320, 320*	*935, 930*	
**Sexting (images or recorded videos)**							
**Did less since lockdown**	69·1 (50·2,83·3)	51·5 (43·1,59·8)	24·4 (18·4,31·6)	14·4 (6·9,27·5)	26·7 (21·9,32·2)	31·8 (28·4,35·5)	<0·0001

**No change since lockdown**	19·1 (8·4,37·8)	20·5 (14·5,28·2)	34·7 (27·9,42·1)	44·7(31·3,58·9)	38·2 (32·8,44·0)	33·5 (30·0,37·2)	
**Did more since lockdown**	11·8 (4·2,28·9)	28·0 (21·1,36·2)	41·0 (33·8,48·6)	40·9 (28·0,55·3)	35·1 (29·8,40·8)	34·7 (31·1,38·4)	
*Denominators (wgt, unwgt)***	*33, 33 ǂ*	*154, 163*	*203, 208*	*57, 53*	*345, 338*	*792, 795*	
**Using video or voice calls to interact with someone sexually**							
**Did less since lockdown**	78·5 (57·0,90·9)	62·9 (52·7,72·0)	24·4 (18·1,32·1)	15·4 (7·5,29·3)	26·8 (21·4,32·9)	34·2 (30·4,38·3)	<0·0001
**No change since lockdown**	11·1 (3·5,30·3)	14·5 (8·9,22·7)	32·9 (25·7,40·9)	38·9 (25·8,53·9)	29·8 (24·2,36·0)	27·8 (24·2,31·7)	
**Did more since lockdown**	10·5 (2·6,34·1)	22·6 (15·1,32·5)	42·7 (35·0,50·8)	45·6 (31·9,60·1)	43·5 (37·1,50·0)	38·0 (34·0,42·2)	
*Denominators (wgt, unwgt)***	*37, 31 ǂ*	*111, 112*	*183, 181*	*56, 50*	*265, 261*	*652, 635*	
**Looking at pornography**							
**Did less since lockdown**	15·8 (10·3,23·5)	12·0 (9·3,15·3)	12·1 (8·4,17·1)	11·5 (6·1,20·9)	10·7 (8·8,13·0)	11·7 (10·3,13·3)	<0·0001
**No change since lockdown**	62·0 (53·3,69·9)	65·6 (61·1,69·9)	50·1 (43·6,56·6)	48·7 (38·0,59·5)	61·7 (58·3,65·0)	60·6 (58·2,62·9)	
**Did more since lockdown**	22·2 (15·9,30·1)	22·4 (18·7,26·6)	37·7 (31·6,44·3)	39·8 (29·6,50·9)	27·6 (24·5,30·8)	27·7 (25·6,29·9)	
*Denominator (unwgt, wgt)***	*158, 167*	*522, 522*	*278, 270*	*91, 94*	*932, 948*	*1981, 2002*	
**Paying for online sexual services (e.g. live streaming)**							
**Did less since lockdown**	··	81·5 (66·5,90·7)	26·8 (18·6,36·9)	··	29 (22·3,36·6)	38·1 (33·0,43·4)	<0·0001
**No change since lockdown**	··	6·6 (2·1,18·7)	30·6 (22·1,40·7)	··	29 (22·5,36·4)	24·9 (20·6,29·9)	
**Did more since lockdown**	··	11·9 (4·8,26·6)	42·6 (32·9,52·9)	··	42·1 (34·8,49·8)	37 (32·1,42·3)	
*Denominator (unwgt, wgt) ***	*25, 25 ǂǂ*	*46, 46 ***	*110, 116*	*24, 27 ǂǂ*	*187, 200*	*392, 415*	

CI=confidence intervals.
∞ 24 participants who identified "in another way" are included in data presented for all participants but excluded from "Men" and "Women". Trans men and trans women are included in data for men and women, respectively.
* Denominator: All respondents (excluding those with no partnered experience ever) who reported the activity since lockdown OR did not do the activity during lockdown and reported a decrease·
** Denominator: All respondents who reported the activity since lockdown OR did not do the activity during lockdown and reported a decrease
ǂ Unweighted denominator <50. Results should be interpreted with caution due to small denominator.
ǂǂ Unweighted denominator <30. Results not shown due to small denominator.

Appendix 6b: Extent and direction of perceived change in frequency of particular physical and virtual sexual activities compared to pre-lockdown, stratified by experience of partnered sex and relationship status since lockdown: Women ∞

Reported partnered sex since lockdown	No		Yes				
	Among those reporting never having had partnered sex % (CI)	Among those reporting partnered sex ever but not since lockdown % (CI)	Among those not in a steady relationship % (CI)	Among those in a steady non-cohabiting relationship % (CI)	Among those in a steady cohabiting relationship % (CI)	All	p-value
**Vaginal, anal and/or oral sex**							
**Did less since lockdown**	··	100	42·3 (35·6,49·3)	46·7 (39·4,54·0)	15·3 (13·5,17·3)	28·6 (26·6,30·6)	<0·0001
**No change since lockdown**	··	0	31·6 (25·5,38·3)	37·7 (31·0,45·0)	70·1 (67·6,72·4)	57·1 (54·8,59·3)	
**Did more since lockdown**	··	0	26·2 (20·5,32·7)	15·6 (11·2,21·4)	14·6 (12·8,16·6)	14·4 (12·9,16·0)	
*Denominator (unwgt, wgt)**	··	*218, 196*	*235, 191*	*214, 178*	*1548, 1496*	*2215, 2061*	
**Other contact with someone's genital area**							
**Did less since lockdown**	··	100	43·0 (35·8,50·4)	45·6 (38·1,53·3)	13·8 (12·0,15·9)	28·7 (26·6,30·9)	<0·0001
**No change since lockdown**	··	0	33·2 (26·5,40·6)	41·5 (34·2,49·2)	72·3 (69·6,74·7)	58·0 (55·7,60·4)	
**Did more since lockdown**	··	0	23·9 (18·2,30·7)	12·9 (8·7,18·9)	13·9 (12·1,16·0)	13·3 (11·7,15·0)	
*Denominator (unwgt, wgt)**	··	*218, 193*	*210, 168*	*194, 159*	*1331, 1267*	*1953, 1787*	
**Masturbation**							
**Did less since lockdown**	24·8 (16·8,35·0)	20·2 (16·1,25·1)	17·3 (12·0,24·2)	17·9 (12·2,25·5)	18·8 (15·9,22·0)	19·3 (17·3,21·6)	<0·0001
**No change since lockdown**	56·6 (45·9,66·7)	64·2 (58·7,69·3)	52·4 (44·3,60·3)	48·4 (39·2,57·7)	64·7 (60·9,68·2)	61·5 (58·8,64·1)	
**Did more since lockdown**	18·7 (11·9,28·1)	15·6 (12·1,19·8)	30·4 (23·6,38·1)	33·7 (25·4,43·1)	16·5 (13·9,19·5)	19·2 (17·2,21·4)	
*Denominator (unwgt, wgt)***	*105, 91*	*356, 323*	*179, 137*	*134, 104*	*765, 682*	*1539, 1337*	
**Using sex toys (by yourself or with someone else**							
**Did less since lockdown**	50·5 (34·9,66·0)	24·4 (18·4,31·5)	18·9 (12·8,26·9)	19·8 (12·7,29·6)	16·6 (13·4,20·4)	20·4 (17·8,23·3)	<0·0001
**No change since lockdown**	32·2 (19·2,48·7)	58·1 (50·5,65·3)	51·3 (42·3,60·3)	55·8 (44·5,66·5)	65·1 (60·6,69·5)	59·5 (56·2,62·8)	
**Did more since lockdown**	17·3 (8·5,32·1)	17·6 (12·7,23·8)	29·8 (22·4,38·4)	24·4 (15·7,35·9)	18·3 (14·9,22·2)	20·1 (17·5,22·9)	
*Denominators (wgt, unwgt)***	*44, 42 ǂ*	*194, 168*	*143, 107*	*95, 74*	*521, 453*	*997, 844*	
**Messaging via dating apps/ online**							
**Did less since lockdown**	45·8 (34·8,57·3)	34·0 (27·6,41·1)	25·9 (18·8,34·5)	17·0 (10·2,27·1)	28·1 (21·1,36·3)	30·6 (27·0,34·5)	0·002
**No change since lockdown**	27·3 (18·4,38·5)	31·6 (25·5,38·5)	29·2 (21·6,38·0)	49·6 (38·2,61·1)	38·7 (30·9,47·2)	34·4 (30·7,38·4)	
**Did more since lockdown**	26·9 (18·0,38·0)	34·4 (28·1,41·2)	45·0 (36·2,54·1)	33·4 (23·6,44·9)	33·2 (25·9,41·4)	35·0 (31·3,38·9)	
*Denominators (wgt, unwgt)***	*75, 91*	*191, 229*	*108, 141*	*69, 89*	*140, 167*	*583, 717*	
**Sexting (images or recorded videos)**							
**Did less since lockdown**	68·9 (52·9,81·4)	53·4 (43·9,62·6)	30·8 (22·5,40·6)	30·0 (20·5,41·6)	28·0 (21·8,35·2)	38·0 (33·8,42·5)	<0·0001
**No change since lockdown**	27·3 (15·5,43·5)	27·3 (19·4,36·9)	27·8 (19·8,37·4)	32·9 (23·1, 44·5)	44·6 (37·3,52·1)	34·6 (30·4,38·9)	

**Did more since lockdown**	3·8 (1·3,10·8)	19·3 (13·2,27·3)	41·4 (31·8,51·8)	37·1 (26·7,48·9)	27·4 (21·1,34·7)	27·4 (23·6,31·6)	
*Denominators (wgt, unwgt)***	*39, 43* ‡	*107, 125*	*87, 117*	*61, 84*	*175, 211*	*469, 580*	
**Using video or voice calls to interact with someone sexually**							
**Did less since lockdown**	··	59·8 (48·0,70·7)	32·2 (22·3,44·1)	30·2 (19·8,43·2)	37·7 (29·3,46·8)	43·4 (38·2,48·8)	<0·0001
**No change since lockdown**	··	18·7 (11·1,29·9)	26·4 (17·1,38·3)	17·2 (9·4,29·5)	34·9 (26·9,43·8)	25·9 (21·5,30·9)	
**Did more since lockdown**	··	21·4 (13·4,32·4)	41·4 (30·3,53·5)	52·6 (39·5,65·3)	27·4 (20·4,35·9)	30·7 (26·0,35·8)	
*Denominators (wgt, unwgt)***	*28, 28* ‡‡	*68, 80*	*71, 82*	*47, 64* ‡	*132, 147*	*346, 401*	
**Looking at pornography**							
**Did less since lockdown**	33·0 (21·5,46·9)	28·0 (21·1,36·0)	18·8 (12·5,27·4)	15·0 (8·5,25·1)	18·9 (15·1,23·3)	21·3 (18·4,24·5)	0·0001
**No change since lockdown**	47·8 (34·5,61·4)	51·5 (43·2,59·7)	45·6 (36·4,55·2)	52·3 (40·4,63·9)	61·5 (56·2,66·5)	55·4 (51·7,59·0)	
**Did more since lockdown**	19·3 (11·0,31·6)	20·6 (14·4,28·5)	35·6 (27·1,45·0)	32·8 (22·6,44·9)	19·7 (15·8,24·3)	23·3 (20·3,26·6)	
*Denominator (unwgt, wgt)***	*66, 59*	*168, 133*	*135, 103*	*89, 68*	*421, 364*	*879, 727*	
**Paying for online sexual services (E.g. live streaming)**							
**Did less since lockdown**	··	··	43·4 (25·6,63·05)	··	35·5 (23·8,49·1)	54·2 (45·6,62·6)	<0·0001
**No change since lockdown**	··	··	12·4 (4·9,27·8)	··	34·2 (22·8,47·8)	18·0 (12·3,25·7)	
**Did more since lockdown**	··	··	44·2 (26·2,63·8)	··	30·4 (20·2,42·9)	27·8 (20·9,35·9)	
*Denominator (unwgt, wgt) ***	*15, 16* ‡‡	*28, 21* ‡‡	*32, 26* ‡	*15, 12* ‡‡	*70, 59*	*160, 134*	

CI=confidence intervals.
∞ 24 participants who identified "in another way" are included in data presented for all participants but excluded from "Men" and "Women". Trans men and trans women are included in data for men and women, respectively.
* Denominator: All respondents (excluding those with no partnered experience ever) who reported the activity since lockdown OR did not do the activity during lockdown and reported a decrease·
** Denominator: All respondents who reported the activity since lockdown OR did not do the activity during lockdown and reported a decrease
‡ Unweighted denominator <50. Results should be interpreted with caution due to small denominator.
‡‡ Unweighted denominator <30. Results not shown due to small denominator.

Mercer CH, *et al. Sex Transm Infect* 2021;0:1–9. doi: 10.1136/sextrans-2021-055210

Appendix 7a: Extent and direction of perceived change in frequency of particular physical and virtual sexual activities compared to pre-lockdown, stratified by experience of partnered sex and relationship status since lockdown: Age-group 18-24 years

Reported partnered sex since lockdown	No		Yes			All	p-value
	Among those reporting never having had partnered sex % (CI)	Among those reporting partnered sex ever but not since lockdown % (CI)	Among those not in a steady relationship % (CI)	Among those in a steady non-cohabiting relationship % (CI)	Among those in a steady cohabiting relationship % (CI)		
**Vaginal, anal and/or oral sex**							
Did less since lockdown	··	100·0	38·4 (30·3,47·2)	45·4 (35·6,55·6)	19·5 (14·4,25·8)	41·4 (37·1,45·9)	<0·0001
No change since lockdown	··	0·0	26·2 (19·1,34·9)	31·9 (23·2,42·1)	47·9 (40·8,55·1)	31·8 (27·8,36·1)	
Did more since lockdown	··	0·0	35·4 (27·4,44·2)	22·7 (15·2,32·4)	32·6 (26·0,40·0)	26·8 (22·9,31·1)	
*Denominator (unwgt, wgt)**	··	*92, 73*	*157, 139*	*110, 86*	*239, 191*	*598, 488*	
**Other contact with someone's genital area**							
Did less since lockdown	··	100·0	36·6 (28·7,45·3)	41·5 (31·8,51·9)	16·6 (11·4,23·4)	39·6 (35·2,44·2)	<0·0001
No change since lockdown	··	0·0	29·2 (21·6,38·0)	37·1 (27·7,47·7)	52·2 (44·6,59·7)	34·9 (30·7,39·4)	
Did more since lockdown	··	0·0	34·2 (26·3,43·2)	21·4 (13·6,31·9)	31·2 (24·5,38·9)	25·5 (21·5,29·9)	
*Denominator (unwgt, wgt) **	··	*96, 73*	*151, 133*	*107, 85*	*221, 180*	*575, 471*	
**Masturbation**							
Did less since lockdown	18·6 (11·1,29·3)	14·1 (8·213,23·2)	20·0 (13·8,28·1)	13·2 (7·5,22·1)	20·4 (14·2,28·5)	17·9 (14·7,21·7)	0·054
No change since lockdown	52·7 (41·5,63·6)	41·8 (32·2,52·0)	32·1 (24·6,40·7)	32·8 (22·8,44·7)	38·8 (30·7,47·7)	39·3 (35·1,43·8)	
Did more since lockdown	28·8 (19·7,39·9)	44·1 (34·23,54·4)	47·9 (39·1,56·9)	53·9 (42·0,65·4)	40·8 (32·3,49·9)	42·7 (38·3,47·3)	
*Denominator (unwgt, wgt) **	*93, 93*	*115, 90*	*150, 135*	*81, 66*	*162, 132*	*601, 516*	
**Using sex toys (by yourself or with someone else)**							
Did less since lockdown	··	38·5 (25·25,53·8)	20·6 (13·2,30·7)	16·9 (8·6,30·5)	27·4 (18·0,39·4)	27·2 (22·0,33·1)	0·015
No change since lockdown	··	29·2 (18·1,43·5)	38·4 (28·7,49·3)	35·1 (22·2,50·6)	42·3 (32·2,53·1)	36·8 (31·2,42·7)	
Did more since lockdown	··	32·3 (20·39,47·1)	41·0 (30·9,51·9)	48·0 (32·5,63·8)	30·3 (21·5,40·9)	36·0 (30·4,42·1)	
*Denominator (unwgt, wgt) ***	*22, 16* ǂǂ	*56, 38*	*105, 88*	*48, 38* ǂ	*114, 84*	*345, 264*	
**Messaging via dating apps/ online**							
Did less since lockdown	48·3 (34·8,62·0)	27·9 (19·27,38·4)	24·2 (16·7,33·7)	17·2 (9·1,30·1)	30·9 (19·8,44·8)	28·9 (24·2,34·1)	0·034
No change since lockdown	26·6 (16·2,40·5)	23·8 (16·0,33·7)	33·2 (24·7,43·1)	38·7 (26·7,52·3)	29·7 (20·0,41·8)	30·3 (25·7,35·3)	
Did more since lockdown	25·1 (15·5,38·0)	48·4 (38·0,58·9)	42·6 (33·2,52·6)	44·1 (31·2,57·9)	39·3 (27·9,52·1)	40·8 (35·8,46·1)	
*Denominator (unwgt, wgt) ***	*64, 53*	*106, 79*	*124, 114*	*61, 48*	*83, 73*	*438, 367*	
**Sexting (images or recorded videos)**							
Did less since lockdown	··	57·5 (43·6,70·2)	25·5 (17·5,35·6)	24·9 (15·1,38·3)	31·9 (21·6,44·2)	35·1 (29·6,40·9)	0·0001
No change since lockdown	··	19·1 (10·5,32·4)	24·5 (16·8,34·3)	26·2 (15·8,40·0)	31·6 (21·2,44·3)	26·5 (21·6,32·1)	

Did more since lockdown	··	23·4 (14·15,36·2)	50·0 (39·6,60·3)	48·9 (35·3,62·7)	36·5 (25·8,48·8)	38·4 (32·8,44·4)	
*Denominator (unwgt, wgt) ***	*29, 26 ǂǂ*	*61, 46*	*112, 100*	*59, 48*	*91, 77*	*352, 297*	
**Using video or voice calls to interact with someone sexually**							
Did less since lockdown	··	68·6 (50·2,82·6)	26·9 (18·2,37·8)	23·0 (13·2,37·0)	26·8 (16·9,39·8)	34·5 (28·6,41·0)	<0·0001
No change since lockdown	··	10·2 (4·0,23·7)	28·1 (19·1,39·2)	25·0 (13·1,42·5)	30·4 (20·2,43·0)	24·4 (19·1,30·5)	
Did more since lockdown	··	21·2 (9·7,40·3)	45·1 (34·4,56·2)	52·0 (36·5,67·1)	42·8 (30·4,56·2)	41·1 (34·7,47·8)	
*Denominator (unwgt, wgt) ***	*23, 24 ǂǂ*	*35, 28 ǂ*	*96, 93*	*49, 41 ǂ*	*72, 65*	*275, 250*	
**Looking at pornography**							
Did less since lockdown	14·2 (8·0,23·9)	21·6 (13·5,32·8)	18·9 (12·4,27·7)	14·4 (7·2,26·7)	19·8 (13·3,28·4)	18·2 (14·7,22·2)	0·38
No change since lockdown	55·2 (43·2,66·6)	40·8 (30·1,52·4)	38·0 (29·3,47·6)	36·3 (24·0,50·7)	43·4 (33·9,53·4)	42·9 (38·1,47·9)	
Did more since lockdown	30·6 (20·7,42·6)	37·6 (27·1,49·5)	43·1 (33·9,52·9)	49·3 (35·4,63·3)	36·8 (27·3,47·5)	38·9 (34·1,44·0)	
*Denominators (unwgt, wgt)***	*82, 82*	*89, 74*	*129, 118*	*59, 50*	*132, 108*	*491, 433*	
**Paying for online sexual services (E.g. live streaming)**							
Did less since lockdown	··	··	28·8 (17·7,43·2)	··	28·5 (15·1,47·2)	37·9 (29·6,47·1)	<0·0001
No change since lockdown	··	··	26·3 (15·9,40·4)	··	23·3 (12·6,39·1)	21·6 (15·1,30·0)	
Did more since lockdown	··	··	44·9 (31·5,59·0)	··	48·2 (32·5,64·2)	40·4 (32·0,49·5)	
*Denominators (wgt, unwgt) ***	*10, 9 ǂǂ*	*13, 10 ǂǂ*	*61, 62*	*18, 18 ǂǂ*	*50, 48*	*152, 146*	

CI=confidence intervals.

* Denominator: All respondents (excluding those with no partnered experience ever) who reported the activity since lockdown OR did not do the activity during lockdown and reported a decrease.

** Denominator: All respondents who reported the activity since lockdown OR did not do the activity during lockdown and reported a decrease

ǂ Unweighted denominator <50. Results should be interpreted with caution due to small denominator.

ǂǂ Unweighted denominator <30. Results not shown due to small denominator

Appendix 7b: Extent and direction of perceived change in frequency of particular physical and virtual sexual activities compared to pre-lockdown, stratified by experience of partnered sex and relationship status since lockdown: Age-group 25-34 years

Reported partnered sex since lockdown	No		Yes			All	p-value
	Among those reporting never having had partnered sex % (CI)	Among those reporting partnered sex ever but not since lockdown % (CI)	Among those not in a steady relationship % (CI)	Among those in a steady non-cohabiting relationship % (CI)	Among those in a steady cohabiting relationship % (CI)		
**Vaginal, anal and/or oral sex**							
Did less since lockdown	··	100·0	39·3 (32·2,46·8)	46·7 (37·5,56·1)	16·4 (14·1,19·1)	29·4 (26·9,32·0)	<0·0001
No change since lockdown	··	0·0	35·6 (28·8,43·1)	37·3 (28·7,46·7)	62·3 (59·0,65·5)	51·1 (48·3,53·9)	
Did more since lockdown	··	0·0	25·1 (19·1,32·2)	16·1 (10·4,24·0)	21·2 (18·6,24·1)	19·5 (17·4,21·8)	
*Denominator (unwgt, wgt)**	··	*124, 114*	*213, 188*	*126, 110*	*997, 912*	*1460, 1324*	
**Other contact with someone's genital area**							
Did less since lockdown	··	100·0	46·6 (38·6,54·8)	45·0 (35·4,55·0)	15·4 (13·0,18·1)	30·1 (27·5,32·9)	<0·0001
No change since lockdown	··	0·0	34·8 (27·4,42·9)	39·5 (30·3,49·4)	64·1 (60·7,67·4)	52·0 (49·1,54·9)	
Did more since lockdown	··	0·0	18·6 (13·2,25·6)	15·5 (9·6,24·1)	20·6 (17·9,23·5)	17·9 (15·8,20·2)	
*Denominator (unwgt, wgt) **	··	*128, 118*	*182, 158*	*113, 100*	*925, 838*	*1348, 1214*	
**Masturbation**							
Did less since lockdown	34·4 (21·9,49·4)	14·4 (10·1,20·1)	11·1 (6·7,17·7)	15·9 (9·5,25·3)	14·8 (12·1,17·9)	15·4 (13·3,17·7)	0·007
No change since lockdown	43·0 (30·0,57·1)	55·4 (47·8,62·8)	55·5 (47·1,63·7)	54·2 (42·9,65·2)	59·5 (55·5,63·5)	57·0 (53·9,60·1)	
Did more since lockdown	22·6 (13·3,35·9)	30·2 (23·6,37·8)	33·4 (26·0,41·7)	29·9 (20·5,41·4)	25·7 (22·3,29·4)	27·7 (25·0,30·5)	
*Denominator (unwgt, wgt) **	*63, 60*	*296, 186*	*173, 146*	*87, 73*	*679, 612*	*1208, 1075*	
**Using sex toys (by yourself or with someone else)**							
Did less since lockdown	··	36·9 (26·3,49·1)	28·1 (19·8,38·2)	19·2 (10·6,32·3)	14·3 (11·0,18·3)	21·3 (18·1,24·8)	<0·0001
No change since lockdown	··	39·7 (29·1,51·3)	48·2 (38·4,58·1)	51·8 (38·2,65·2)	59·6 (54·3,64·7)	53·6 (49·5,57·6)	
Did more since lockdown	··	23·4 (14·7,35·0)	23·8 (16·4,33·1)	29·0 (18·0,43·0)	26·1 (21·7,31·1)	25·2 (21·8,28·9)	
*Denominator (unwgt, wgt) ***	*26, 24 ǂǂ*	*83, 62*	*122, 107*	*60, 47*	*418, 367*	*709, 606*	
**Messaging via dating apps/ online**							
Did less since lockdown	53·5 (37·3,69·0)	34·1 (26·4,42·8)	27·8 (20·5,36·4)	16·3 (8·2,29·9)	29·1 (22·7,36·5)	30·8 (26·8,35·0)	0·043
No change since lockdown	22·0 (11·3,38·4)	30·3 (22·7,39·1)	35·9 (27·9,44·8)	49·8 (35·1,64·5)	35·7 (28·7,43·3)	34·5 (30·4,38·9)	
Did more since lockdown	24·5 (13·4,40·7)	35·6 (27·5,44·5)	36·3 (28·2,45·3)	33·9 (21·2,49·4)	35·2 (28·3,42·9)	34·7 (30·6,39·1)	
*Denominator (unwgt, wgt) ***	*44, 37 ǂ*	*149, 138*	*151, 131*	*51, 43*	*192, 181*	*587, 531*	
**Sexting (images or recorded videos)**							
Did less since lockdown	··	45·7 (34·8,57·1)	28·1 (20·1,37·8)	11·3 (5·2,23·0)	26·1 (20·6,32·5)	30·4 (26·3,34·9)	<0·0001
No change since lockdown	··	24·8 (16·1,36·1)	33·6 (25·1,43·2)	53·3 (38·7,67·4)	39·8 (33·4,46·6)	36·0 (31·6,40·5)	

Mercer CH, *et al. Sex Transm Infect* 2021;0:1–9. doi: 10.1136/sextrans-2021-055210

Did more since lockdown	··	29·5 (20·2,41·0)	38·3 (29·2,48·4)	35·4 (22·8,50·4)	34·1 (27·9,40·9)	33·6 (29·3,38·2)	
*Denominator (unwgt, wgt) ** *	*20, 19* ǂǂ	*88, 79*	*126, 110*	*52, 45*	*240, 227*	*526, 479*	
**Using video or voice calls to interact with someone sexually**							
Did less since lockdown	··	51·4 (38·4,64·2)	22·3 (14·2,33·2)	13·7 (6·7,26·2)	29·2 (22·6,36·9)	32·4 (27·6,37·5)	<0·0001
No change since lockdown	··	17·4 (9·7,29·3)	37·5 (26·9,49·3)	40·1 (26·1,56·0)	28·3 (21·7,36·0)	28·9 (24·3,34·1)	
Did more since lockdown	··	31·2 (20·0,45·1)	40·3 (29·5,52·0)	46·1 (31·5,61·4)	42·5 (34·9,50·4)	38·7 (33·6,44·1)	
*Denominator (unwgt, wgt) ** *	*15, 16* ǂǂ	*67, 59*	*89, 86*	*46, 41* ǂ	*177, 169*	*394, 370*	
**Looking at pornography**							
Did less since lockdown	30·2 (17·2,47·4)	14·0 (9·4,20·3)	10·5 (6·2,17·4)	9·1 (4·1,19·2)	12·5 (9·7,16·0)	13·2 (11·0,15·8)	0·079
No change since lockdown	44·4 (29·7,60·2)	53·6 (45·0,62·0)	54·8 (45·8,63·4)	60·5 (47·7,72·0)	57·7 (52·9,62·4)	56·0 (52·4,59·4)	
Did more since lockdown	25·4 (14·4,40·7)	32·5 (24·7,41·3)	34·7 (26·8,43·6)	30·4 (20·1,43·1)	29·8 (25·6,34·3)	30·8 (27·6,34·2)	
*Denominators (unwgt, wgt)** *	*48, 51* ǂ	*160, 150*	*159, 139*	*70, 61*	*495, 469*	*932, 870*	
**Paying for online sexual services (E.g. live streaming)**							
Did less since lockdown	··	··	31·1 (19·0,46·5)	··	20·6 (13·9,29·5)	34·8 (28·2,41·9)	<0·0001
No change since lockdown	··	··	26·2 (15·3,41·3)	··	30·7 (22·3,40·7)	25·2 (19·3,32·1)	
Did more since lockdown	··	··	42·7 (28·8,57·8)	··	48·6 (39·1,58·3)	40·1 (33·3,47·3)	
*Denominators (wgt, unwgt) ** *	*12, 13* ǂǂ	*23, 19* ǂǂ	*51, 51*	*12, 12* ǂǂ	*118, 117*	*216, 213*	

CI=confidence intervals.
* Denominator: All respondents (excluding those with no partnered experience ever) who reported the activity since lockdown OR did not do the activity during lockdown and reported a decrease.
** Denominator: All respondents who reported the activity since lockdown OR did not do the activity during lockdown and reported a decrease
ǂ Unweighted denominator <50. Results should be interpreted with caution due to small denominator.
ǂǂ Unweighted denominator <30. Results not shown due to small denominator

Appendix 7c: Extent and direction of perceived change in frequency of particular physical and virtual sexual activities compared to pre-lockdown, stratified by experience of partnered sex and relationship status since lockdown: Age-group 35-44 years

Reported partnered sex since lockdown	No		Yes			All	p-value
	Among those reporting never having had partnered sex % (CI)	Among those reporting partnered sex ever but not since lockdown % (CI)	Among those not in a steady relationship % (CI)	Among those in a steady non-cohabiting relationship % (CI)	Among those in a steady cohabiting relationship % (CI)		
**Vaginal, anal and/or oral sex**							
Did less since lockdown	··	100·0	35·0 (25·4,46·0)	40·4 (27·0,55·4)	17·6 (15·0,20·5)	27·8 (25·0,30·7)	<0·0001
No change since lockdown	··	0·0	40·3 (30·2,51·3)	43·7 (29·9,58·5)	66·6 (63·0,69·9)	57·1 (53·9,60·2)	
Did more since lockdown	··	0·0	24·7 (16·5,35·3)	15·9 (7·6,30·4)	15·9 (13·4,18·8)	15·1 (12·9,17·6)	
*Denominator (unwgt, wgt)**	··	*97, 105*	*92, 93*	*48, 51 ǂ*	*795, 875*	*1032, 1124*	
**Other contact with someone's genital area**							
Did less since lockdown	··	100·0	33·9 (24·1,45·4)	35·6 (22·1,51·8)	17·0 (14·3,20·2)	27·5 (24·5,30·6)	<0·0001
No change since lockdown	··	0·0	44·2 (33·1,55·8)	45·0 (29·4,61·6)	67·5 (63·7,71·1)	57·9 (54·4,61·3)	
Did more since lockdown	··	0·0	21·9 (13·9,32·9)	19·4 (9·3,36·2)	15·4 (12·7,18·6)	14·7 (12·3,17·3)	
*Denominator (unwgt, wgt) **	··	*90, 95*	*84, 84*	*39, 42 ǂ*	*681, 746*	*894, 967*	
**Masturbation**							
Did less since lockdown	18·1 (9·3,32·2)	14·9 (10·2,21·2)	13·0 (7·0,23·0)	9·7 (3·1,26·2)	14·7 (11·7,18·2)	14·6 (12·2,17·3)	0·724
No change since lockdown	65·8 (50·8,78·2)	68·0 (60·57,74·66)	64·0 (51·8,74·6)	66·3 (47·8,81·0)	61·5 (56·9,66·0)	63·6 (60·1,67·0)	
Did more since lockdown	16·2 (7·9,30·1)	17·1 (12·2,23·5)	23·0 (14·4,34·7)	24·0 (12·0,42·3)	23·8 (20·0,28·1)	21·8 (19·0,25·0)	
*Denominator (unwgt, wgt) **	*52, 51*	*195, 198*	*75, 72*	*31, 34 ǂ*	*499, 536*	*852, 891*	
**Using sex toys (by yourself or with someone else)**							
Did less since lockdown	··	30·5 (20·33,43·0)	18·7 (9·7,32·8)	··	21·5 (16·6,27·5)	24·1 (19·8,28·9)	0·032
No change since lockdown	··	56·9 (44·23,68·7)	62·2 (46·5,75·7)	··	58·2 (51·5,64·7)	57·9 (52·5,63·0)	
Did more since lockdown	··	12·6 (6·362,23·5)	19·2 (9·6,34·6)	··	20·2 (15·4,26·2)	18·1 (14·3,22·6)	
*Denominator (unwgt, wgt) ***	*10, 11 ǂǂ*	*68, 70*	*46, 42 ǂ*	*16, 19 ǂǂ*	*241, 257*	*381, 398*	
**Messaging via dating apps/ online**							
Did less since lockdown	··	42·7 (32·95,53·06)	15·7 (8·3,27·8)	··	23·3 (16·4,32·0)	29·5 (24·5,35·0)	0·023
No change since lockdown	··	37·1 (27·96,47·3)	42·3 (29·5,56·2)	··	42·7 (33·8,52·1)	40·9 (35·3,46·6)	
Did more since lockdown	··	20·2 (13·26,29·4)	42·0 (29·4,55·7)	··	34·0 (25·9,43·2)	29·7 (24·8,35·1)	
*Denominator (unwgt, wgt) ***	*26, 28 ǂǂ*	*105, 111*	*59, 59*	*16, 18 ǂǂ*	*129, 134*	*335, 350*	
**Sexting (images or recorded videos)**							
Did less since lockdown	··	41·0 (29·0,54·2)	21·4 (12·3,34·7)	··	27·4 (20·3,35·9)	31·3 (26·0,37·3)	0·020
No change since lockdown	··	28·3 (17·9,41·8)	44·0 (31·0,57·9)	··	39·8 (31·7,48·6)	37·4 (31·7,43·5)	

Did more since lockdown	··	30·7 (20·1,43·8)	34·5 (22·7,48·6)	··	32·8 (25·1,41·5)	31·3 (25·9,37·2)	
*Denominator (unwgt, wgt) ** *	*14, 13 ǂǂ*	*66, 64*	*58, 57*	*15, 16 ǂǂ*	*144, 151*	*297, 301*	
**Using video or voice calls to interact with someone sexually**							
Did less since lockdown	··	57·7 (42·5,71·6)	31·4 (19·0,47·2)	··	33·1 (24·0,43·6)	40·2 (33·5,47·3)	0·009
No change since lockdown	··	24·3 (13·5,39·7)	29·4 (17·3,45·5)	··	34·0 (24·9,44·5)	29·7 (23·6,36·7)	
Did more since lockdown	··	18·0 (9·18,32·4)	39·1 (25·4,54·8)	··	32·9 (23·9,43·5)	30·1 (24·0,36·9)	
*Denominator (unwgt, wgt) ** *	*12, 14 ǂǂ*	*46, 50 ǂ*	*46, 47 ǂ*	*11, 12 ǂǂ*	*96, 108*	*211, 230*	
**Looking at pornography**							
Did less since lockdown	22·9 (11·2,41·3)	16·4 (11,2·62)	9·7 (4·6,19·5)	··	14·9 (11·5,19·1)	15·4 (12·7,18·6)	0·43
No change since lockdown	56·1 (38·2,72·5)	63·2 (54·6,71·0)	55·1 (42·2,67·3)	··	60·1 (54·6,65·3)	60·2 (56·1,64·2)	
Did more since lockdown	21·0 (9·8,39·4)	20·5 (14·3,28·4)	35·2 (24·0,48·3)	··	25·0 (20·5,30·2)	24·4 (20·9,28·2)	
*Denominators (unwgt, wgt)** *	*36, 35 ǂ*	*158, 160*	*67, 65*	*20, 23 ǂǂ*	*366, 393*	*647, 678*	
**Paying for online sexual services (E.g. live streaming)**							
Did less since lockdown	··	··	··	··	43·3 (31·3,56·1)	44·0 (34·6,53·8)	0·036
No change since lockdown	··	··	··	··	30·9 (20·3,43·9)	25·8 (18·2,35·4)	
Did more since lockdown	··	··	··	··	25·9 (16·5,38·1)	30·2 (22·1,39·7)	
*Denominators (wgt, unwgt) ** *	*9, 9 ǂǂ*	*15, 14 ǂǂ*	*20, 20 ǂǂ*	*5, 6 ǂǂ*	*65, 73*	*114, 122*	

CI=confidence intervals.
* Denominator: All respondents (excluding those with no partnered experience ever) who reported the activity since lockdown OR did not do the activity during lockdown and reported a decrease.
** Denominator: All respondents who reported the activity since lockdown OR did not do the activity during lockdown and reported a decrease
ǂ Unweighted denominator <50. Results should be interpreted with caution due to small denominator.
ǂǂ Unweighted denominator <30. Results not shown due to small denominator

Appendix 7d: Extent and direction of perceived change in frequency of particular physical and virtual sexual activities compared to pre-lockdown, stratified by experience of partnered sex and relationship status since lockdown: Age-group 45-59 years

**Reported partnered sex since lockdown**	**No**		**Yes**				
	**Among those reporting never having had partnered sex % (CI)**	**Among those reporting partnered sex ever but not since lockdown % (CI)**	**Among those not in a steady relationship % (CI)**	**Among those in a steady non-cohabiting relationship % (CI)**	**Among those in a steady cohabiting relationship % (CI)**	**All**	**p-value**
**Vaginal, anal and/or oral sex**							
Did less since lockdown	··	100·0	43·6 (33·1,54·6)	53·9 (41·2,66·2)	14·5 (12·3,16·9)	28·6 (26·1,31·3)	<0·0001
No change since lockdown	··	0·0	42·1 (31·8,53·1)	41·3 (29·6,54·2)	76·9 (74·0,79·6)	63·7 (60·8,66·4)	
Did more since lockdown	··	0·0	14·3 (8·2,23·9)	4·8 (1·6,13·4)	8·6 (6·8,10·7)	7·7 (6·3,9·5)	
*Denominator (unwgt, wgt)**	··	*153, 156*	*95, 88*	*66, 69*	*936, 1004*	*1250, 1315*	
**Other contact with someone's genital area**							
Did less since lockdown	··	100·0	50·1 (39·1,61·2)	51·8 (38·8,64·4)	12·8 (10·7,15·4)	29·3 (26·6,32·2)	<0·0001
No change since lockdown	··	0·0	34·5 (24·8,45·6)	44·5 (32·2,57·6)	78·9 (75·7,81·7)	63·2 (60·2,66·2)	
Did more since lockdown	··	0·0	15·4 (8·8,25·4)	3·7 (1·0,12·9)	8·3 (6·5,10·6)	7·5 (6·0,9·3)	
*Denominator (unwgt, wgt) **	··	*150, 151*	*91, 83*	*62, 63*	*793, 842*	*1096, 1139*	
**Masturbation**							
Did less since lockdown	13·3 (7·2,23·0)	15·5 (12·2,19·5)	14·4 (7·8,25·3)	18·6 (9·0,34·5)	13·7 (10·7,17·3)	14·6 (12·5,16·9)	<0·0001
No change since lockdown	77·6 (66·9,85·6)	75·8 (71·3,79·9)	58·0 (45·6,69·4)	51·4 (34·8,67·7)	74·2 (69·9,78·1)	73·3 (70·4,76·0)	
Did more since lockdown	9·1 (4·4,17·9)	8·6 (6·2,11·9)	27·6 (18·0,39·7)	30·1 (16·4,48·5)	12·1 (9·4,15·5)	12·2 (10·3,14·4)	
*Denominator (unwgt, wgt) **	*88, 88*	*414, 420*	*77, 68*	*40, 39* ǂ	*476, 473*	*1095, 1088*	
**Using sex toys (by yourself or with someone else)**							
Did less since lockdown	··	36·0 (26·53,46·6)	23·8 (12·0,41·6)	··	16·7 (12·1,22·5)	24·2 (20·0,29·0)	0·004
No change since lockdown	··	55·3 (44·52,65·5)	56·1 (38·6,72·1)	··	71·0 (64·3,76·9)	63·1 (57·8,68·0)	
Did more since lockdown	··	8·8 (4·255,17·2)	20·2 (9·6,37·1)	··	12·3 (8·4,17·7)	12·7 (9·6,16·8)	
*Denominator (unwgt, wgt) ***	*20, 21* ǂǂ	*96, 96*	*39, 34* ǂ	*21, 22* ǂǂ	*223, 225*	*399, 397*	
**Messaging via dating apps/ online**							
Did less since lockdown	··	47·1 (37·82,56·7)	27·3 (15·4,43·7)	··	29·2 (19·6,41·1)	37·9 (32·0,44·1)	0·229
No change since lockdown	··	33·2 (24·94,42·6)	39·9 (25·5,56·3)	··	48·8 (37·3,60·5)	40·6 (34·6,46·8)	
Did more since lockdown	··	19·7 (13·17,28·4)	32·8 (20·1,48·6)	··	21·9 (13·9,32·9)	21·6 (16·9,27·1)	
*Denominator (unwgt, wgt) ***	*24, 27* ǂǂ	*120, 118*	*48, 40* ǂ	*20, 21* ǂǂ	*85, 74*	*297, 279*	
**Sexting (images or recorded videos)**							
Did less since lockdown	··	65·1 (52·81,75·7)	31·8 (16·0,53·1)	··	25·0 (15·6,37·5)	46·1 (38·8,53·6)	<0·0001
No change since lockdown	··	21·0 (12·6,32·8)	37·4 (20·2,58·6)	··	54·5 (42·1,66·4)	35·6 (28·8,43·1)	

Mercer CH, *et al. Sex Transm Infect* 2021;0:1–9. doi: 10.1136/sextrans-2021-055210

Did more since lockdown	··	13·9 (7·5,24·3)	30·8 (16·0,51·1)	··	20·5 (12·4,31·9)	18·3 (13·4,24·5)	
*Denominator (unwgt, wgt) ** *	*14, 16 ǂǂ*	*74, 73*	*30, 24 ǂ*	*12, 11 ǂǂ*	*77, 67*	*207, 191*	
**Using video or voice calls to interact with someone sexually**							
Did less since lockdown	··	76·6 (61·5,87·0)	30·9 (16·0,51·2)	··	32·3 (20·8,46·4)	50·0 (41·6,58·3)	<0·0001
No change since lockdown	··	8·6 (3·3,20·8)	24·2 (11·7,43·4)	··	38·5 (26·5,52·1)	23·3 (17·1,30·9)	
Did more since lockdown	··	14·8 (6·8,29·3)	44·9 (27·2,64·1)	··	29·2 (18·7,42·6)	26·8 (20·1,34·8)	
*Denominator (unwgt, wgt) ** *	*10, 12 ǂǂ*	*45, 44 ǂ*	*32, 28 ǂ*	*9, 10 ǂǂ*	*64, 57*	*160, 152*	
**Looking at pornography**							
Did less since lockdown	17·1 (9·0,30·1)	13·3 (9·7,18·0)	18·8 (10·0,32·6)	9·9 (3·2,27·1)	9·2 (6·5,12·9)	12·0 (9·8,14·6)	<0·0001
No change since lockdown	76·5 (63·3,86·0)	73·6 (67·9,78·6)	50·2 (36·5,63·9)	41·6 (24·7,60·8)	74·6 (69·6,79·0)	71·5 (68·0,74·7)	
Did more since lockdown	6·4 (2·4,16·0)	13·1 (9·491,17·8)	31·0 (19·8,45·1)	48·4 (30·0,67·3)	16·2 (12·6,20·6)	16·6 (14·0,19·5)	
*Denominators (unwgt, wgt)** *	*63, 63*	*287, 275*	*60, 52*	*32, 30 ǂ*	*364, 346*	*806, 765*	
**Paying for online sexual services (E.g. live streaming)**							
Did less since lockdown	··	··	··	··	··	68·1 (56·2,78·1)	<0·0001
No change since lockdown	··	··	··	··	··	16·7 (9·8,27·0)	
Did more since lockdown	··	··	··	··	··	15·2 (8·4,26·0)	
*Denominators (wgt, unwgt) ** *	*9, 10 ǂǂ*	*24, 25 ǂǂ*	*11, 10 ǂǂ*	*5, 4 ǂǂ*	*24, 23 ǂǂ*	*73, 72*	

CI=confidence intervals.
* Denominator: All respondents (excluding those with no partnered experience ever) who reported the activity since lockdown OR did not do the activity during lockdown and reported a decrease.
** Denominator: All respondents who reported the activity since lockdown OR did not do the activity during lockdown and reported a decrease
ǂ Unweighted denominator <50. Results should be interpreted with caution due to small denominator.
ǂǂ Unweighted denominator <30. Results not shown due to small denominator

Appendix 8: Percentages, crude and adjusted odds ratios (OR) for perceiving a decrease and an increase (each relative to no change) in sexual frequency compared to pre-lockdown, according to experience of partnered sex/relationship status since lockdown, gender, and age-group

	Reported no change/ stayed the same sexual frequency since lockdown		Reported *decreased* sexual frequency since lockdown				Reported *increased* sexual frequency since lockdown			
	**% (95% CI)**	***Denominator (unweighted-weighted)⁑***	**% (95% CI)**	**OR (95% CI)**	**RAG§ Adjusted OR (95%CI)**	***Denominator (unweighted-weighted) ⁑***	**% (95% CI)**	**OR (95% CI)**	**RAG§ Adjusted OR (95% CI)**	***Denominator (unweighted-weighted) ⁑***
**Partnered sex and relationship status since lockdown**										
Had partnered sex since lockdown and in a steady cohabiting relationship	56·8 (54·9-58·7)	*1629, 1665*	23·9 (22·3-25·6)	1·00	1·00	*731, 701*	19·3 (17·9-20·9)	1·00	1·00	*573, 567*
Had partnered sex since lockdown and in a steady non-cohabiting relationship	36·7 (31·4-42·3)	*127, 117*	50·3 (44·7-55·9)	3·25 (2·50-4·24)	2·75 (2·08-3·63)	*176, 160*	13·0 (9·8-17·2)	1·04 (1·49-2·57)	0·76 (0·52-1·13)	*49, 42* **
Had partnered sex since lockdown and not in a steady relationship	33·3 (29·0-37·9)	*164, 167*	44·5 (40·0-49·1)	3·17 (2·51-4·01)	2·64 (2·06-3·38)	*255, 223*	22·2 (18·7-26·2)	1·96 (1·49-2·57)	1·47 (1·10-1·97)	*131, 111*
Not had partnered sex since lockdown	66·0 (63·3-68·5)	*948, 975*	33·3 (30·8-35·9)	1·20 (1·03-1·39)	1·29 (1·10-1·50)	*499, 492*	0·7 (0·4-1·5)	Excluded from model†		*9, 11* *
**Age-group**										
45-59	67·9 (65·7-70·1)	*1273, 1384*	25·4 (23·4-27·5)	1·00	1·00	*512, 517*	6·7 (5·6-8·0)	1·00	1·00	*125, 136*
35-44	57·9 (55·0-60·8)	*730, 790*	28·3 (25·7-30·9)	1·31 (1·10-1·55)	1·31 (1·10-1·57)	*363, 385*	13·8 (11·9-16·0)	2·43 (1·87-3·15)	1·78 (1·35-2·34)	*174, 189*
25-34	47·6 (44·9-50·2)	*787, 709*	32·3 (29·9-34·9)	1·82 (1·55-2·14)	1·74 (1·47-2·07)	*532, 482*	20·1 (18·1-22·3)	4·30 (3·39-5·46)	2·72 (2·11-3·51)	*332, 300*
18-24	39·3 (35·4-43·4)	*274, 230*	40·1 (36·2-44·2)	2·73 (2·20-3·38)	2·51 (1·96-3·21)	*299, 235*	20·6 (17·4-24·1)	5·32 (3·95-7·16)	4·03 (2·87-5·67)	*145, 120*
**Gender**										
Men	55·9 (53·8-57·9)	*1478, 1533*	31·0 (29·2-32·9)	1·00	1·00	*857, 851*	13·2 (11·8-14·6)	1·00	1·00	*343, 361*
Women	57·8 (55·8-59·7)	*1576, 1569*	28·1 (26·4-29·9)	0·88 (0·77-1·00)	0·87 (0·76- 1·00)	*845, 764*	14·1 (12·8-15·5)	1·03 (0·87-1·22)	1·14 (0·94-1·38)	*432, 382*

⁑ Denominator: All respondents who reported partnered sex ever (excluding those missing/ 'did not answer' question on change in frequency)
§ Adjusted for RAG - relationship status, age and gender
†Excluded from analysis as this group reported no partnered sex since lockdown, therefore would not have the opportunity to report an increase.

Appendix 9: Percentages, crude and adjusted odds ratios (OR) for perceiving a decrease and an increase (each relative to no change) in sexual satisfaction compared to pre-lockdown, according to partnered sex/relationship status, gender and age-group

	**Reported no change/ stayed the same sexual satisfaction since lockdown**		**Reported *decreased* sexual satisfaction since lockdown**					**Reported *increased* sexual satisfaction since lockdown**				
	**% (95% CI)**	***Denominator (unweighted, weighted)* ⁑**	**% (95% CI)**	**OR (95% CI)**	**RAG§ Adjusted OR (95%CI)**	**RAG§ and sexual satisfaction Adjusted OR (95%CI)**	***Denominator (unweighted, weighted)* ⁑**	**% (95% CI)**	**OR (95% CI)**	**RAG§ Adjusted OR (95%CI)**	**RAG§ and sexual satisfaction Adjusted OR (95%CI)**	***Denominator (unweighted, weighted)* ⁑**
**Partnered sex and relationship status since lockdown**												
Had partnered sex since lockdown and in a steady cohabiting relationship	66·6 (64·7-68·4)	*1909, 1948*	16·5 (15·2-18·0)	1·00	1·00	1·00	*509, 484*	16·9 (15·5-18·4)	1·00	1·00	1·00	*509, 495*
Had partnered sex since lockdown and in a steady non-cohabiting relationship	55·7 (50·0-61·2)	*191, 177*	25·5 (20·9-30·7)	1·84 (1·38-2·47)	1·54 (1·14-2·09)	0·88 (0·62-1·24)	*88, 81*	18·8 (14·9-23·5)	1·33 (0·97-1·82)	1·03 (0·74-1·44)	1·35 (0·94-1·94)	*71, 60*
Had partnered sex since lockdown and not in a steady relationship	43·7 (39·1-48·3)	*225, 218*	31·0 (26·9-35·4)	2·86 (2·24-3·64)	2·29 (1·77-2·97)	1·73 (1·29-2·32)	*177, 155*	25·4 (21·6-29·5)	2·29 (1·78-2·94)	1·65 (1·25-2·18)	1·64 (1·18-2·27)	*146, 127*
Not had partnered sex since lockdown	64·9 (62·2-67·4)	*934, 963*	32·7 (30·2-35·3)	2·03 (1·73-2·37)	2·25 (1·91-2·66)	2·19 (1·81-2·65)	*488, 485*	2·5 (1·8-3·5)	0·15 (0·11-0·22)	0·18 (0·13-0·26)	0·36 (0·25-0·54)	*37, 37***
**Age-group**												
45-59	74·6 (72·5-76·6)	*1407, 1523*	18·8 (17·1-20·7)	1·00	1·00	1·00	*376, 384*	6·6 (5·6-7·9)	1·00	1·00	1·00	*130, 135*
35-44	63·9 (61·1-66·6)	*807, 871*	23·7 (21·4-26·3)	1·47 (1·23-1·77)	1·67 (1·38-2·03)	1·62 (1·31-2·00)	*301, 324*	12·4 (10·6-14·4)	2·18 (1·68-2·83)	1·77 (1·35-2·31)	1·48 (1·10-1·99)	*157, 168*
25-34	56·2 (53·6-58·8)	*921, 838*	24·4 (22·2-26·7)	1·72 (1·44-2·05)	1·97 (1·63-2·38)	1·69 (1·36-2·10)	*407, 364*	19·4 (17·4-21·6)	3·29 (3·08-4·92)	2·81 (2·19-3·59)	2·00 (1·53-2·61)	*319, 289*
18-24	45·5 (41·5-49·6)	*327, 269*	31·5 (27·8-35·4)	2·74 (2·19-3·44)	2·64 (2·05-3·41)	2·18 (1·59-3·00)	*227, 186*	23·0 (19·7-26·6)	5·69 (4·28-7·55)	4·39 (3·20-6·02)	2·95 (2·04-4·25)	*168, 136*
**Gender**												
Men	55·9 (58·9-62·9)	*1630, 1683*	25·2 (23·5-27·0)	1·00	1·00	1·00	*691, 697*	13·9 (12·5-15·3)	1·00	1·00	1·00	*368, 383*
Women (including trans women	66·8 (64·9-68·6)	*1824, 1810*	20·5 (19·0-22·1)	0·74 (0·65-0·85)	0·75 (0·65-0·87)	0·76 (0·65-0·90)	*615, 556*	12·7 (11·5-14·0)	0·84 (0·71-0·99)	0·86 (0·72-1·03)	0·78 (0·64-0·96)	*405, 344*
**Sexual frequency**												
Stayed the same since lockdown	83·1 (81·7-84·5)	*2504, 2576*	11·1 (10·0-12·3)	1·00		1·00	*355, 344*	5·8 (5·0-6·7)	1·00		1·00	*190, 180*
Decreased since lockdown	38·5 (36·0-41·0)	*647, 616*	53·7 (51·1-56·3)	10·46 (8·89-12·30)		10·11 (8·49-12·03)	*902, 860*	7·9 (6·6-9·4)	2·93 (2·26-3·79)		2·13 (1·69-2·91)	*134, 126*
Increased since lockdown	38·3 (34·7-42·1)	*286, 284*	4·8 (3·5-6·7)	0·95 (0·64-1·39)		1·03 (0·69-1·54)	*38, 36**	56·8 (53·0-60·6)	21·25 (16·95-26·64)		14·27 (11·21-18·18)	*447, 421*

⁑ Denominator: All respondents who reported partnered sex ever (excluding those missing/ 'did not answer' question on change in satisfaction)
§ Adjusted for RAG - relationship status, age and gender

Mercer CH, *et al. Sex Transm Infect* 2021;0:1–9. doi: 10.1136/sextrans-2021-055210
